# A unified model for Duchenne muscular dystrophy gene involvement in cancer: context‐dependent tumour suppression and oncogenicity

**DOI:** 10.1002/2211-5463.70109

**Published:** 2025-08-20

**Authors:** Lee Machado, Leanne Jones, Sonika Divakar, Michael Naidoo, Karen Anthony

**Affiliations:** ^1^ Centre for Physical Activity and Life Sciences University of Northampton UK; ^2^ Department of Cancer and Genomic Sciences, School of Medical Sciences, College of Medicine and Health University of Birmingham UK

**Keywords:** cancer, Dp71, Duchenne, dystrophin, dystrophin‐associated protein complex

## Abstract

Evidence implicates the Duchenne muscular dystrophy gene (*DMD*) in tumorigenesis, but survival trends are inconsistent. To resolve this, we conducted a comprehensive global analysis of *DMD* expression and survival outcomes across 33 tumour types using bulk RNA sequencing data from The Cancer Genome Atlas. We examined the impact of total *DMD*, individual transcript and dystrophin‐associated protein complex (DAPC) gene expression levels on overall survival using Kaplan–Meier analysis, Cox proportional hazard modelling and pathway analysis. *DMD* expression was significantly associated with survival in nine cancers after Bonferroni correction (α = 0.0015), with high expression linked to either improved or worsened outcomes depending on cancer type. The most abundant *DMD* transcript, Dp71ab, mirrored total *DMD* trends, distinguishing two tumour groups with opposing survival associations. Hierarchical clustering suggests these divergent effects may be linked to a subset of signalling and adhesion‐related DAPC components. Our findings indicate that *DMD* does not act uniformly as an oncogene or tumour suppressor. Instead, we propose a context‐dependent dual model whereby high *DMD* expression is tumour suppressive in aggressive cancers and oncogenic in less aggressive tumours.

## Graphical abstract

We propose a context‐dependent model where the Duchenne muscular dystrophy (DMD) gene acts as a tumour suppressor in aggressive tumours and as an oncogene in less aggressive ones. We propose this model as a unified framework to explain the opposing survival associations with *DMD* expression and to guide experimental exploration of the dual role of *DMD* in cancer.

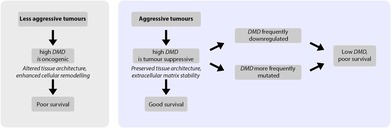

**Abbreviations**
BRCA: breast invasive carcinoma
DAPC: dystrophin‐associated protein complex
DEG: differentially expressed gene
*DMD*
: Duchenne muscular dystrophy
Dp: dystrophin protein
ECM: extracellular matrix
FDR: false discovery rate
FPKM‐UQ: fragments per kilobase million upper quartile
GDC: genomic data commons
GO: gene ontology
HNSCC: head and neck squamous cell carcinoma
HPV: human papillomavirus
HR: hazard ratio
KEGG: Kyoto encyclopedia of genes and genomes
KIRP: kidney renal papillary cell carcinoma
LAML: acute myeloid leukaemia
LGG: low‐grade glioma
LUAD: lung adenocarcinoma
MMP: matrix metalloprotease
PAAD: pancreatic adenocarcinoma
READ: rectum adenocarcinoma
TCGA: The Cancer Genome Atlas
THYM: thymoma
UVM: uveal melanoma

The Duchene muscular dystrophy gene (*DMD*) is named after the clinical condition of the same name and is one of the largest genes in the human genome, comprised of 79 exons spanning 2 Mb on the short arm of chromosome X (ChrX (p21.2‐p21.1)). [Correction added on 9 September 2025 after first online publication: The number of exons has been updated to “79” from “97”]. It resides within a known fragile site [1, 2] and encodes a large 427 KDa dystrophin protein. As part of the dystrophin‐associated protein complex (DAPC), dystrophin bridges the inner cytoskeleton to the extracellular matrix. Dystrophin and the DAPC have unique structural and biochemical roles that are tissue dependent. The canonical role of the DAPC is mechanical, where it stabilises the plasma membrane of striated muscle cells, but it also plays a role in cell signalling [3, 4, 5]. Mutations in many DAPC and DAPC‐associated protein encoding genes cause muscular dystrophy, highlighting its importance in muscle [6], but intriguingly, mutations and alterations in their expression are also associated with numerous types of cancer (including sarcomas, leukaemia's, lymphomas, nervous system tumours, melanomas and carcinomas), recently reviewed by us [2]. *DMD* downregulation is shown to correlate with age of onset, staging and survival in some tumours but gene expression and survival trends vary across others [7, 8, 9, 10, 11, 12].

The *DMD* gene contains seven independent alternative promoters producing multiple dystrophin protein variants, and splice isoforms thereof, which contribute to the complexity of dystrophin biology [13, 14]. Most notably dystrophin protein (Dp) 71, Dp71, is ubiquitously expressed, predominant in the adult brain and alternatively spliced (at exons 71 and 78) to produce four major isoforms that appear to have tissue‐specific expression and functions [14]. The roles of these Dp71 isoforms in membrane stabilisation and cell signalling remain considerably less well‐characterised compared to full‐length dystrophin. It is unclear whether each of these isoforms interact with the DAPC in a manner analogous to the full‐length protein, or whether such interactions result in comparable structural or signalling outcomes across different cell types. Previous studies have shown the normal balance of *DMD* gene products is disrupted in cancer and have linked Dp71 expression to tumorigenesis, but the findings are conflicting, with some suggesting it is oncogenic and others indicating a tumour‐suppressive role [2, 7, 8, 9, 10, 11, 12, 15, 16, 17, 18]. To address these discrepancies, we conducted a comprehensive bioinformatic analysis of *DMD* expression and its prognostic significance across 33 tumour types. Our study examines how *DMD*, its gene variants, and DAPC gene expression are linked to patient survival outcomes and aims to theorise why survival trends differ by cancer type.

## Materials and methods

All data preprocessing and analyses were conducted using the r statistical Software (version 4.0.3; R Foundation for Statistical Computing, Vienna, Austria) and/or graphpad prism 10 (GraphPad Software, LLC, San Diego, CA, USA) unless otherwise stated.

### Data acquisition

Clinical and transcriptomic data for 33 tumour types were obtained from The Cancer Genome Atlas (TCGA) through the Genomic Data Commons (GDC) portal. Transcriptomic data included normalised RNA‐seq data using the FPKM‐UQ (Fragments Per Kilobase Million Upper Quartile) workflow, and isoform expression data retrieved from the GDC Legacy Archive. FPKM‐UQ files were available as tab delimited files with the Ensembl gene IDs in the first column and the expression values in the second. The r/bioconductor package tcgabiolinks [19] version 2.24 was used employing GDCquery(), GDCdownload() and GDCprepare() functions and using *data.catagory* as ‘*Transcriptome profiling*’, *data.type* as ‘Gene Expression quantification’ and *workflow.type* as ‘HTSeq – FPKM‐UQ’. Nonprimary tumours were filtered out during preprocessing.

### Gene expression and survival analysis

To analyse the association of *DMD* gene expression (and DAPC gene expression) with patient survival outcomes, RNAseq data from 33 tumour types from the Genomic Data Commons (GDC) were imported into rstudio using an r tcgabiolinks library [19]. Associated harmonised clinical data were also imported into rstudio. *DMD/*DAPC RNAseq gene expression was linked to patient clinical data to perform survival analysis. High versus low *DMD/*DAPC expressing patients were dichotomised using cut point selection using the R library maxstat. maxstat uses maximally selected rank statistics (smethod = LogRank) to evaluate a simple estimated cut point. Simulation was done using conditional Monte Carlo with B = 9999 replications [20]. Kaplan–Meier survival curves were generated using the survival r package or in graphpad, and differences in survival were assessed via the log‐rank test. For univariate hazard modelling, Cox proportional hazards regression was applied using *DMD* or DAPC gene expression as a sole covariate. Hazard ratios (HR) and 95% confidence intervals (CI) were calculated for each tumour type, focussing on the nine cancers with significant survival differences after Bonferroni correction (α = 0.0015). Results were visualised through forest plots and Kaplan–Meier curves and unweighted pair group method with arithmetic mean hierarchical clustering analysis using a Euclidean distance matrix with the hclust Ward D2 method. Cancers were subsequently grouped according to *DMD* expression survival trends, and the two cohorts compared using the GDC Data Portal Cohort Builder and Analysis Center tools: Cohort Comparison and ProteinPaint available at: https://portal.gdc.cancer.gov/analysis_page?app=.

### Transcript‐specific analysis

To assess the prognostic importance of specific *DMD* transcripts, survival analyses were repeated with patient cohorts stratified by isoform expression. Isoform expression data from the GDC legacy archive data were extracted using the r/bioconductor package tcgabiolinks [19] version 2.24 using GDCquery(), GDCdownload() and GDCprepare() functions for primary tumour *samples.types* as well as using *data.type* as ‘Isoform expression quantification’ and *file.type* as ‘normalized’. This pipeline used mapsplice [21] to do the alignment and RSEM to perform the quantification [22]. Output files contained UCSC isoform identifiers (curated from the UCSC Table browser), which were used to convert them to specific *DMD* gene products for processing and survival analysis. Hazard ratios for these genes were calculated using univariate Cox modelling. Only transcripts with sufficient read coverage and quantification confidence were included to ensure robust expression estimates and avoid artefacts from low‐abundance or poorly annotated isoforms.

### Differential gene expression and functional pathway analysis

Differential gene expression (DEG) analysis and functional enrichment analysis of DEGs was performed using idep, which integrates 63 r/bioconductor packages for pathway analysis [23, 24]. DEG analysis was conducted between high and low *DMD*‐expressing tumours using the limma r package. Genes with a false discovery rate (FDR) < 0.1 and a fold change > 2 were identified as significant. For pathway analysis, Gene Ontology (GO) terms (biological processes, molecular functions and cellular components) and Kyoto Encyclopedia of Genes and Genomes (KEGG) pathways were assessed.

## Results

### *DMD* expression is significantly associated with survival outcomes across at least nine tumour types

**Fig. 1:**
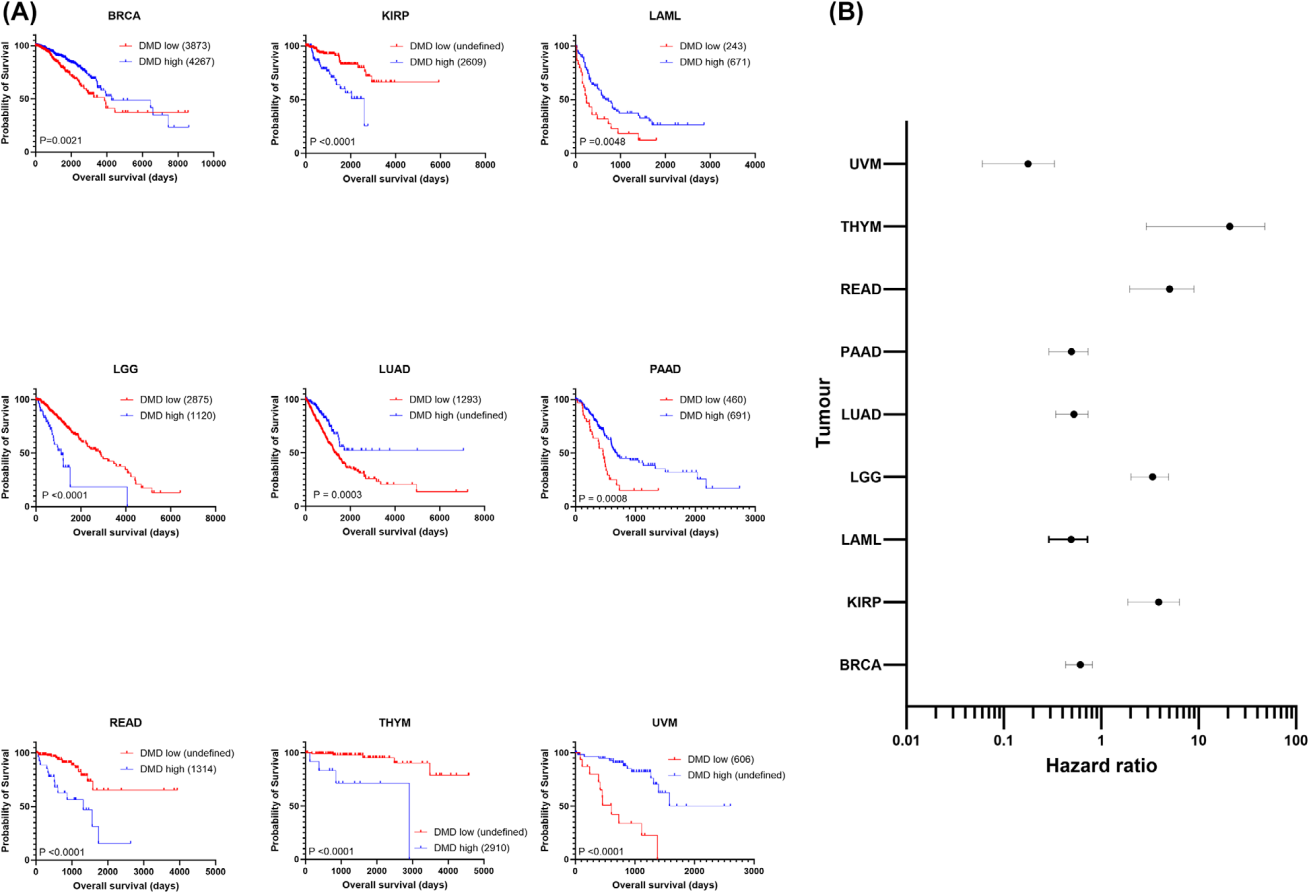
*DMD* expression is significantly associated with survival across tumour types (A) TCGA RNAseq data from nine TCGA cancer cases were dichotomised into high (blue) and low (red) *DMD* expressing groups and survival analysis performed in graphpad using the log‐rank test. Numbers in brackets are median overall survival times in days. (B) Forest plot revealing significant log‐rank hazard ratios illustrated with 95% confidence intervals in selected TCGA tumours. UVM *n* = 80, THYM *n* = 121, READ *n* = 177, PAAD *n* = 182, LUAD *n* = 594, LGG *n* = 529, LAML *n* = 151, KIRP *n* = 321, BRCA *n* = 1222.

Applying an outcome‐based cut‐point approach, survival analysis was employed for 33 TCGA tumours, dichotomising patients into high or low expressing groups (Fig. S1). Of the 33 tumour types examined, nine had significant differences in survival outcomes (Log‐Rank test) after Bonferroni correction (Fig. 1A). These were as follows: breast invasive carcinoma (BRCA, *P* = 0.0001), kidney renal papillary cell carcinoma (KIRP, *P* < 0.001), acute myeloid leukaemia (LAML, *P* = 0.0006), low‐grade glioma (LGG, *P* < 0.0001), lung adenocarcinoma (LUAD, *P* = 0.0003), pancreatic adenocarcinoma (PAAD, *P* = 0.0008), rectum adenocarcinoma (READ, *P* < 0.0001), thymoma (THYM, *P* < 0.0001) and uveal melanoma (UVM, *P* < 0.0001). For BRCA, LAML, LUAD, PAAD, and UVM, patient overall survival was better in those patients with high total *DMD* tumour RNA expression. Conversely, in KIRP, LGG, READ and THYM, high expression of *DMD* was associated with worse survival outcomes. As an example, and shown previously using a different bioinformatic pipeline [10], median survival of LGG patients with high expression of *DMD* was 1120 days compared with patients with low tumour expression of *DMD* who lived for a median of 2875 days (2.57‐fold increase in overall survival time and a nearly 5‐year difference in survival). To better quantify the strength of these associations, we generated a forest plot of univariate hazard ratios (HR) with confidence intervals (Fig. 1B). THYM had the highest risk of poor survival (HR 11.8, 95% CI: 2.9 to 47.8) and UVM had the lowest risk of poor survival (HR 0.14, 95% CI: 0.06 to 0.33), though with wide confidence intervals in both cases.

### Dp71ab and Dp40 are predominant *DMD* transcripts in solid tumours

**Fig. 2:**
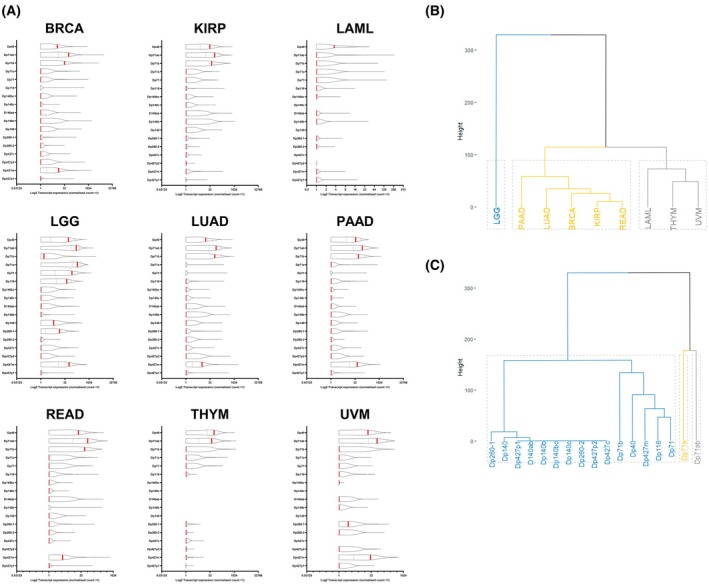
Expression of individual *DMD* gene transcripts in selected TCGA cancers. (A) Normalised counts were Log2 transformed. Red bars represent median values; dashed lines represent 95% confidence intervals. (B) Tumour‐based dendrogram cluster analysis. (C) *DMD* transcript‐based dendrogram cluster analysis. Three clusters were specified for both dendrograms, using Euclidean distance as a distance metric and the Ward D2 clustering algorithm. Dendrograms were generated in r (https://github.com/lrmacha/TCGA).

To determine whether the above findings can be attributed to specific *DMD* transcripts, we investigated the expression of 17 *DMD* transcripts across the nine tumours of interest (Fig. 2A). The results show that Dp71ab (an isoform lacking *DMD* exons 71 and 78) is a predominant transcript across all but one tumour type (LAML). LAML unusually only expresses Dp40 and at weaker levels than for other tumours where Dp40 is also largely predominant. Most tumours express multiple transcripts, with Dp71 variants consistently amongst the highest. Interestingly, only three tumours (THYM, LAML and KIRP) appear to lack full‐length dystrophin transcript expression: Dp427c (cortical promoter), Dp427m (muscle promoter) or Dp427l (lymphocyte promoter). Dp427m is the most predominant full‐length dystrophin transcript, but its expression is variable. UVM uniquely expresses Dp260‐1. LGG exhibited the broadest profile and forms its own distinct cluster expressing nine different transcripts. These findings underscore the tissue specificity of *DMD* transcript expression and their emerging functional diversity. Our results suggest that Dp71ab may be of relevance for tumourigenesis given its predominant expression and the fact that it clusters separately from other transcripts, including Dp40 (Fig. 2C).

### Dp71ab is a key prognostic factor across multiple tumours

**Fig. 3:**
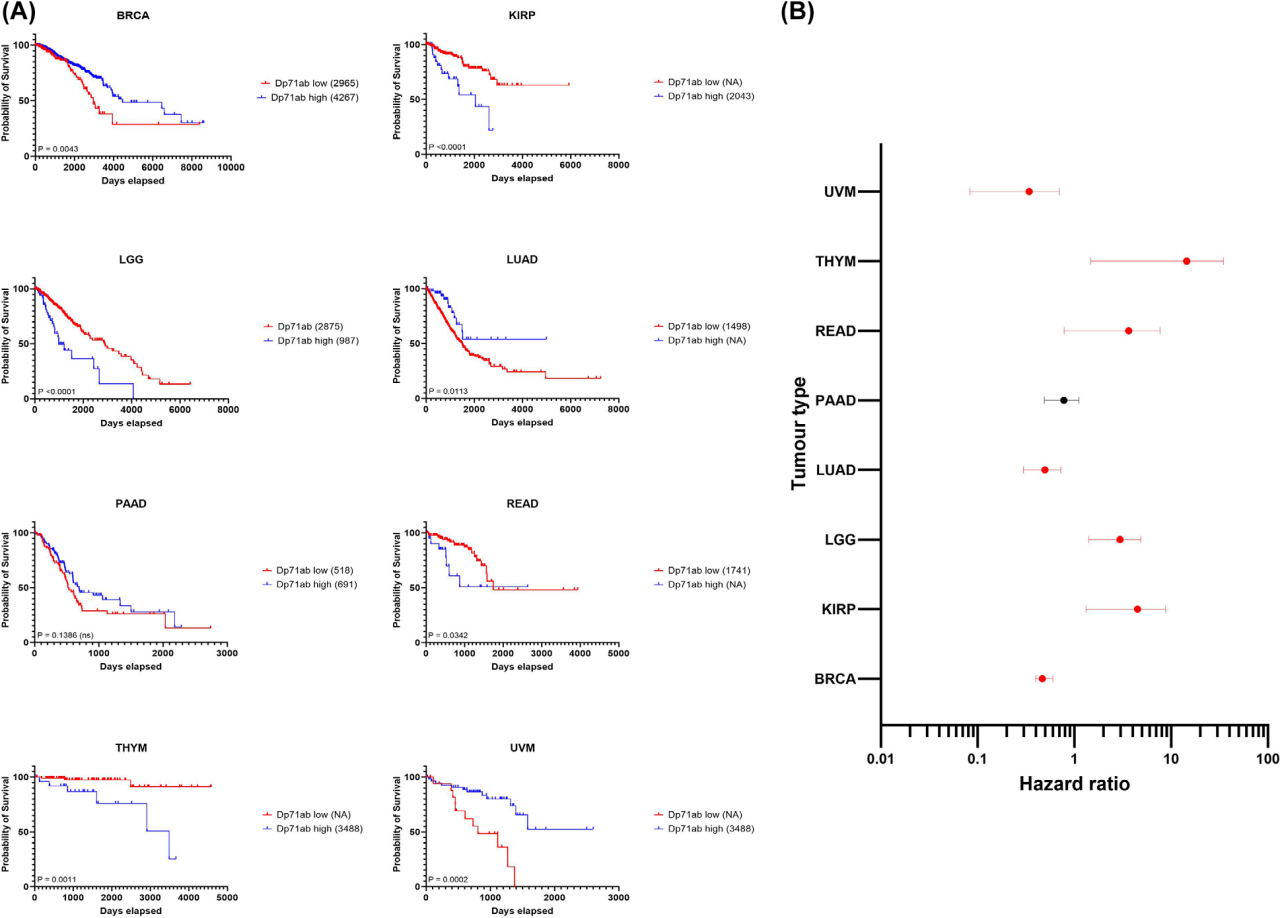
Dp71ab expression is broadly associated with survival across tumour types (A) TCGA RNAseq data from nine TCGA cancer cases were dichotomised into high (blue) and low (red) Dp71ab expressing groups, and survival analysis was performed in graphpad using the log‐rank test. Numbers in brackets are median overall survival times in days. (B) Forest plot revealing log‐rank hazard ratios in selected TCGA tumours illustrated with 95% confidence intervals. UVM *n* = 80, THYM *n* = 121, READ *n* = 177, PAAD *n* = 182, LUAD *n* = 594, LGG *n* = 529, KIRP *n* = 321, BRCA *n* = 1222. Red indicates significance.

Given the expression of multiple *DMD* transcripts in various tumour tissues, we next repeated our survival analysis to determine which gene product(s) are most strongly linked to overall survival. The direction of hazard and survival trends across (and sometimes within) cancer types varies when comparing high versus low expression of different *DMD* gene products (Fig. S2). We focussed our survival analysis on Dp71ab expression given it is the most predominant *DMD* transcript for the largest number of cancers and has a recognised involvement in tumorigenesis [2, 7, 8, 9, 10, 12, 15, 16]. LAML was excluded from analysis due to undetectable Dp71ab expression; out of the eight remaining tumours with significant differences in overall survival after Bonferroni correction, PAAD was the only one that did not exhibit significantly different survival outcomes based on Dp71ab expression (Fig. 3). In line with total *DMD*, high Dp71ab expression in THYM, READ, LGG and KIRP is indicative of poor survival, whilst in UVM, LUAD and BRCA, it is predictive of good survival. This suggests that the effect of *DMD* expression on survival across several tumour types may be attributed to Dp71ab expression.

### Hazard ratio profiling of DAPC genes reveals tumour‐specific prognostic clusters in TCGA data

**Fig. 4:**
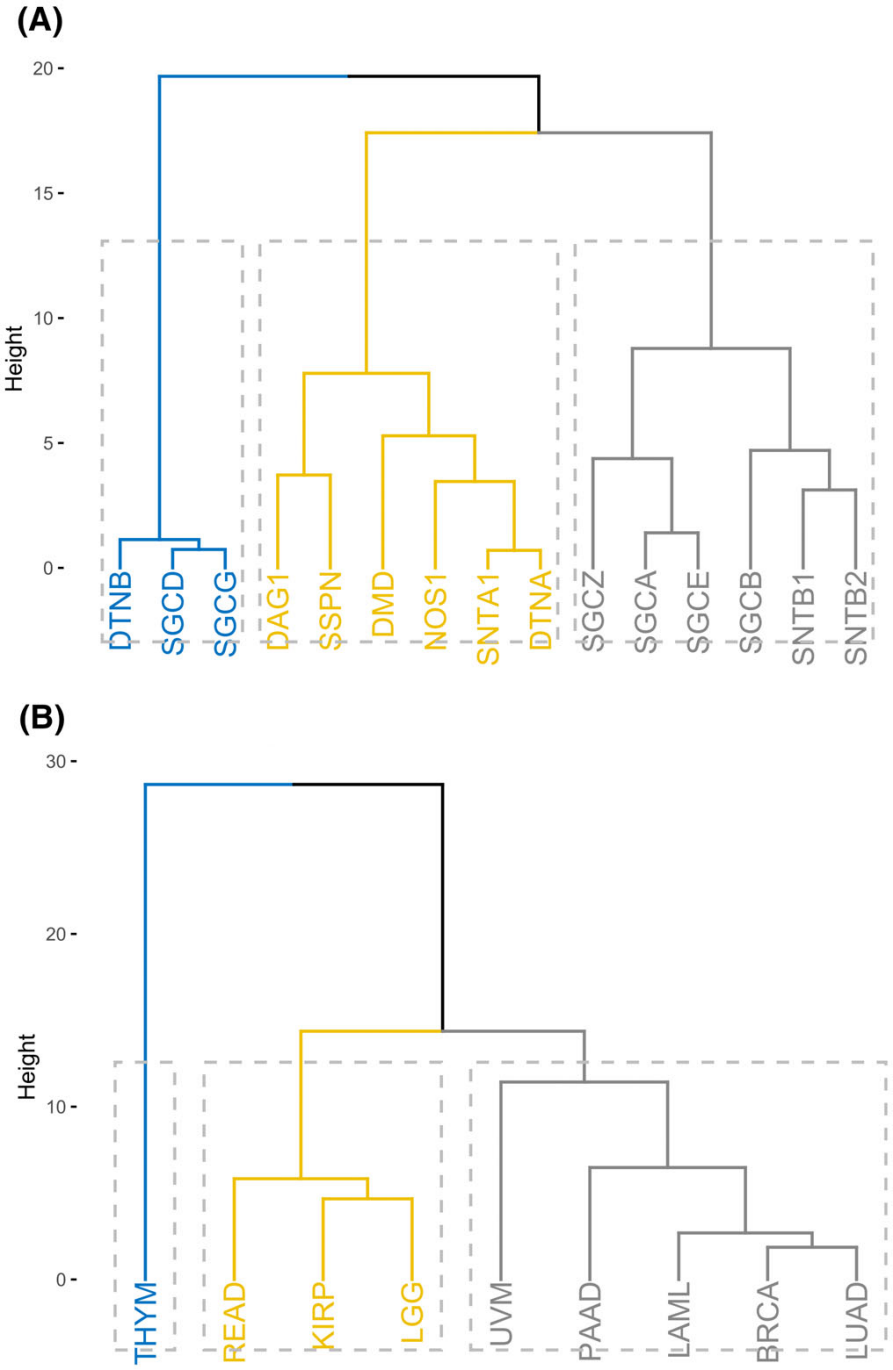
Cluster dendrograms based on DAPC gene hazard ratios. (A) DAPC gene‐based dendrogram cluster analysis of tumours. (B) Tumour‐based dendrogram cluster analysis of DAPC genes. Significant univariate hazard ratio values were used. Three clusters were specified for both dendrograms, using Euclidean distance as a distance metric and the Ward D2 clustering algorithm. Dendrograms were generated in r (https://github.com/lrmacha/TCGA).

To investigate whether other DAPC genes exhibit similar prognostic patterns to *DMD/*Dp71ab, we adopted a candidate approach by profiling the hazard ratios for 14 additional DAPC genes across the nine tumour types with significant *DMD*‐associated survival differences (Fig. 4; Fig. S3). Among the nine tumour types examined, LGG uniquely exhibited statistically significant hazard ratios for all DAPC genes, with nine associated with increased hazard and six linked to reduced hazard. Across the other tumour types, no consistent trends emerged; however, in LUAD and BRCA, high expression of DAPC genes was predominantly protective, with only two (LUAD) or three (BRCA) DAPC genes significantly associated with increased hazard (Fig. S3). To further explore these patterns, hierarchical clustering was performed using hazard ratio values. Clustering by DAPC genes identified three distinct groups (Fig. 4A). The first cluster contained genes encoding two sarcoglycans (gamma and delta) and dystrobrevin beta; the second cluster contained sarcospan, dystrophin, nNOS, dystrobrevin alpha, alpha syntrophin and dystroglycan. The third cluster contained sarcoglycans (alpha, beta, zeta and epsilon) and beta syntrophin 1 and 2 (Fig. 4A). When tumours were clustered based on tumour type instead, three distinct groups emerged, which included two major clusters and a third cluster containing only THYM. The middle cluster contained READ, KIRP and LGG, and the largest cluster contained UVM, PAAD, LAML, BRCA and LUAD (Fig. 4B). This analysis identifies two major tumour clusters and distinct gene clusters, highlighting tumour‐ and gene‐specific variations in prognostic hazard ratio profiles across the nine cancers. These results show that *DMD* groups with a subset of DAPC genes (known for their signalling and adhesion‐related roles) however, unlike *DMD*, individual DAPC gene expression patterns do not clearly separate into two prognostic groups, suggesting more varied or context‐specific roles within tumour progression.

### Distinct pathway enrichment profiles in tumours stratified by *DMD* expression

**Fig. 5:**
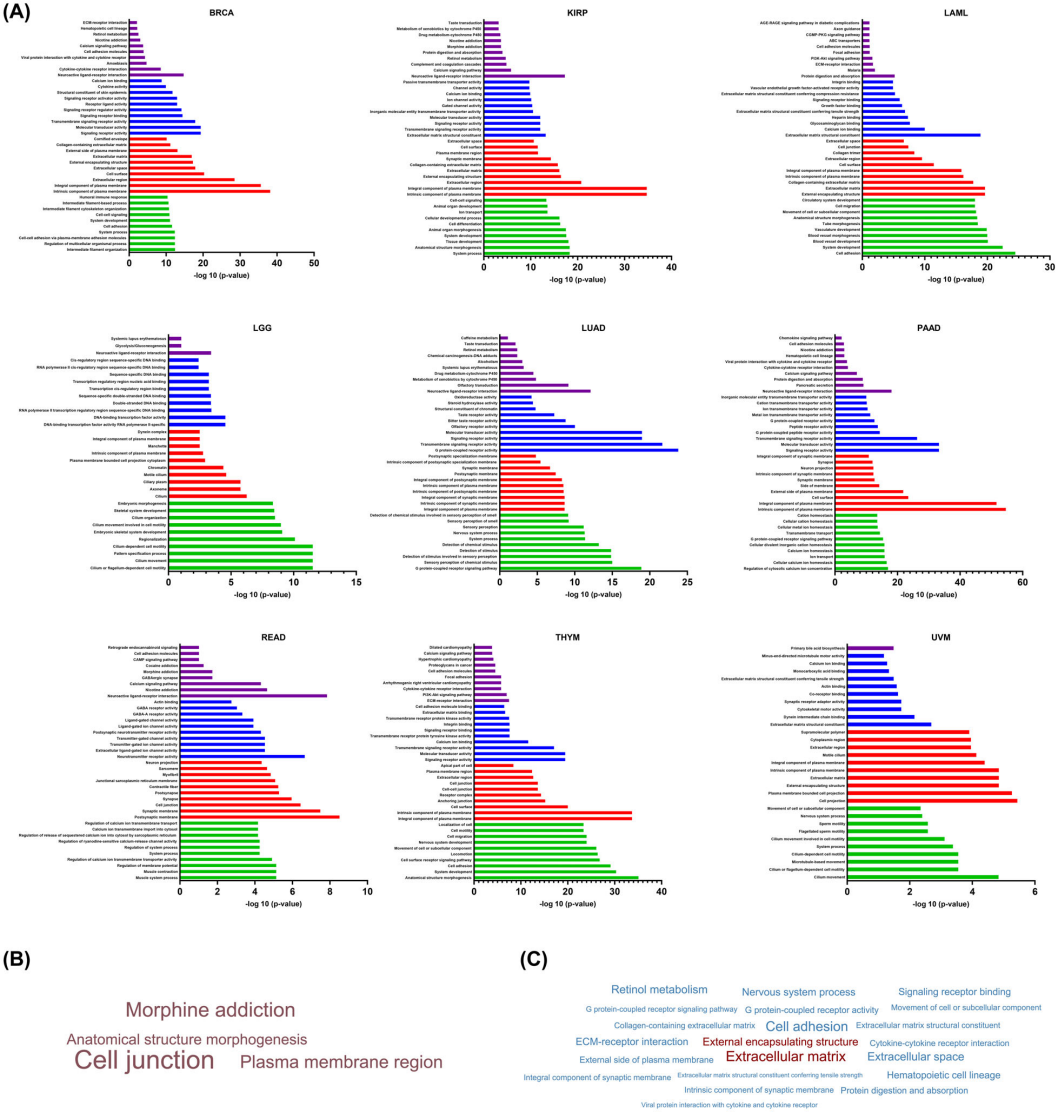
Enrichment analysis of the differentially expressed genes upon high versus low *DMD* expression in selected TCGA tumours. (A) Enrichment analysis against Gene Ontology for Molecular Function (GO MF; Blue), Cellular Component (GO CC; Red), Biological Process (GO BP; Green) and Kyoto Encyclopedia of Genes and Genomes (KEGG; Purple) are shown for the top 10 most significantly increased terms. (B) Sentence cloud depicting uniquely enriched GO and KEGG terms across the tumour dataset where high *DMD* is associated with worse outcomes (i.e. KIRP, LGG, READ and THYM). (C) Sentence cloud depicting uniquely enriched GO and KEGG terms across the tumour dataset where low *DMD* is associated with worse outcomes (i.e. BRCA, LAML, LUAD, PAAD and UVM). Sentence clouds were generated in r (https://github.com/lrmacha/TCGA).

To aid future investigation of functional role(s) for the *DMD* gene across tumour types a pathway analysis of DEGs was undertaken in cases comparing high verses low *DMD* expression. idep was used to identify differentially expressed genes using the deseq2 method [25]. To examine the functional annotations of the DEGs, enrichment analysis gene ontology [GO] biological processes, cellular component, molecular function, and KEGG was explored for the differentially expressed genes (Fig. 5A). The top upregulated GO and KEGG terms highlight key roles in membrane processes, cell signalling, cell communication and extracellular interactions. Enrichment in ‘integral component of plasma membrane’, ‘extracellular matrix structural constituent’ and ‘G protein‐coupled receptor activity’ indicates the importance of membrane structure and receptor‐mediated pathways. Processes including ‘cell adhesion’, ‘focal adhesion’ and ‘extracellular matrix (ECM)‐receptor interaction’ emphasise importance of cell–cell and cell‐matrix communication, supported by ‘extracellular matrix remodelling’. ‘PI3K‐Akt signalling’, ‘cytokine‐cytokine receptor interaction’ and ‘cell migration’ also highlight active intracellular signalling, immune responses and motility. Neural activity is also reflected in ‘synaptic membrane’ and ‘neuron projection’, indicating dynamic cellular interactions and signalling.

To determine commonalities across the group of tumours where high *DMD* expression is linked to improved survival (the *DMD*‐suppressor group, suggesting a tumour‐suppressive role) versus those where high *DMD* expression is associated with poorer survival (the *DMD*‐oncogenic group, indicating an oncogenic role), we identified the recurrent GO and KEGG terms that are uniquely enriched in each group (Fig. 5B,C). In the group of tumours where high *DMD* expression correlates with poorer survival (Fig. 5B), unique enrichment of pathways including ‘anatomical structure morphogenesis’, ‘cell junction’ and ‘plasma membrane region’ point towards enhanced cellular remodelling and dynamic morphogenic processes in high *DMD* versus low *DMD* cases. These pathways are commonly associated with increased cell plasticity, motility and altered tissue architecture, which may contribute to local tumour progression and poor prognosis. In contrast, in the group with high *DMD* expression linked to improved survival (Fig. 5C), unique enrichment of terms such as ‘extracellular matrix’, ‘ECM‐receptor interaction’ and ‘cell adhesion’ emphasise the preservation of extracellular matrix integrity and cell–cell communication in high *DMD* versus low *DMD* cases. Since loss of ECM integrity and adhesion is a well‐recognised trigger for increased invasiveness and metastatic spread, we propose that in these tumours, high *DMD* expression serves as a safeguard, counteracting intrinsic aggressiveness. Conversely, in the *DMD*‐oncogenic group, the association of high *DMD* with pathways promoting dynamic cellular remodelling and motility suggests that these tumours might be less reliant on ECM stability and thus may be inherently less invasive. We therefore hypothesised that tumours in the *DMD*‐suppressor group (where high *DMD* is beneficial) may possess a higher intrinsic potential for invasion and metastasis, which is counteracted by high *DMD* expression, while those in the *DMD*‐oncogenic group may be less aggressive and/or invasive.

### *DMD* exhibits tumour suppressive behaviour in more aggressive cancers and oncogenic behaviour in less aggressive cancers

To test our hypothesis and proposed classification of tumours into aggressive versus less aggressive groups, we used a combination of widely accepted clinical and molecular indicators of tumour aggressiveness. These included median overall survival, metastatic potential, recurrence rates, therapeutic resistance and the burden of somatic alterations (mutations and/or copy number variations), based on publicly available data from the TCGA via the GDC Data Portal and supported by published literature. Tumours exhibiting multiple markers of poor prognosis were designated as ‘aggressive’, while those with comparatively better clinical outcomes and lower genomic instability were grouped as ‘less aggressive’. This classification is summarised in Table 1 and served as the foundation for our downstream comparisons of *DMD*‐related survival trends and mutation profiles.

**Table 1:** *DMD*‐associated cancers categorised against aggressiveness criteria.

	UVM	PAAD	LUAD	LAML	BRCA	SARC	HNSCC	LGG	THYM	READ	KIRP	MESO
	*DMD* suppressive (aggressive)							*DMD* oncogenic (less aggressive)				
Metastatic potential	High, liver metastases common	High, frequent early spread to liver/lung	High	Spreads systemically	Metastatic subtypes common	High, especially osteosarcoma	Lymph node and distant metastases common	Rarely metastasizes outside central nervous system	Distant metastasis is rare	Metastasis occurs but at later stage	Less frequent than clear cell renal cell carcinoma	Locally invasive but rarely distant metastasis
Overall survival	Poor, aggressive cases have < 1 year survival	Poor, ~ 10% 5‐year survival	Poor, late‐stage survival low	Poor, aggressive subtypes have short survival	Variable, aggressive subtypes have poor survival	Poor, depends on subtype	Poor, HPV negative cases worse	Better than glioblastoma	Generally good prognosis	Moderate outcomes	Moderate survival	Poor, median survival ~ 12 months^a^
Therapy resistance	Resistant to chemotherapy	Highly resistant	Resistant to some therapies	Relapses common	Therapy resistant subtypes exist	Chemotherapy resistance is common	Resistant to chemotherapy/radiotherapy	Somewhat therapy responsive	Usually therapy sensitive	Therapy responsive in many cases	Can respond to targeted therapy	Therapy resistant^a^
Tumour mutational burden	High	High	High	High chromosomal instability	Genetic instability in aggressive cases	High genetic heterogeneity	High	Lower mutational burden than glioblastoma	Low	Moderate	Lower mutational burden	Low

^a^ Inconsistent with the proposed model.

**Fig. 6:**
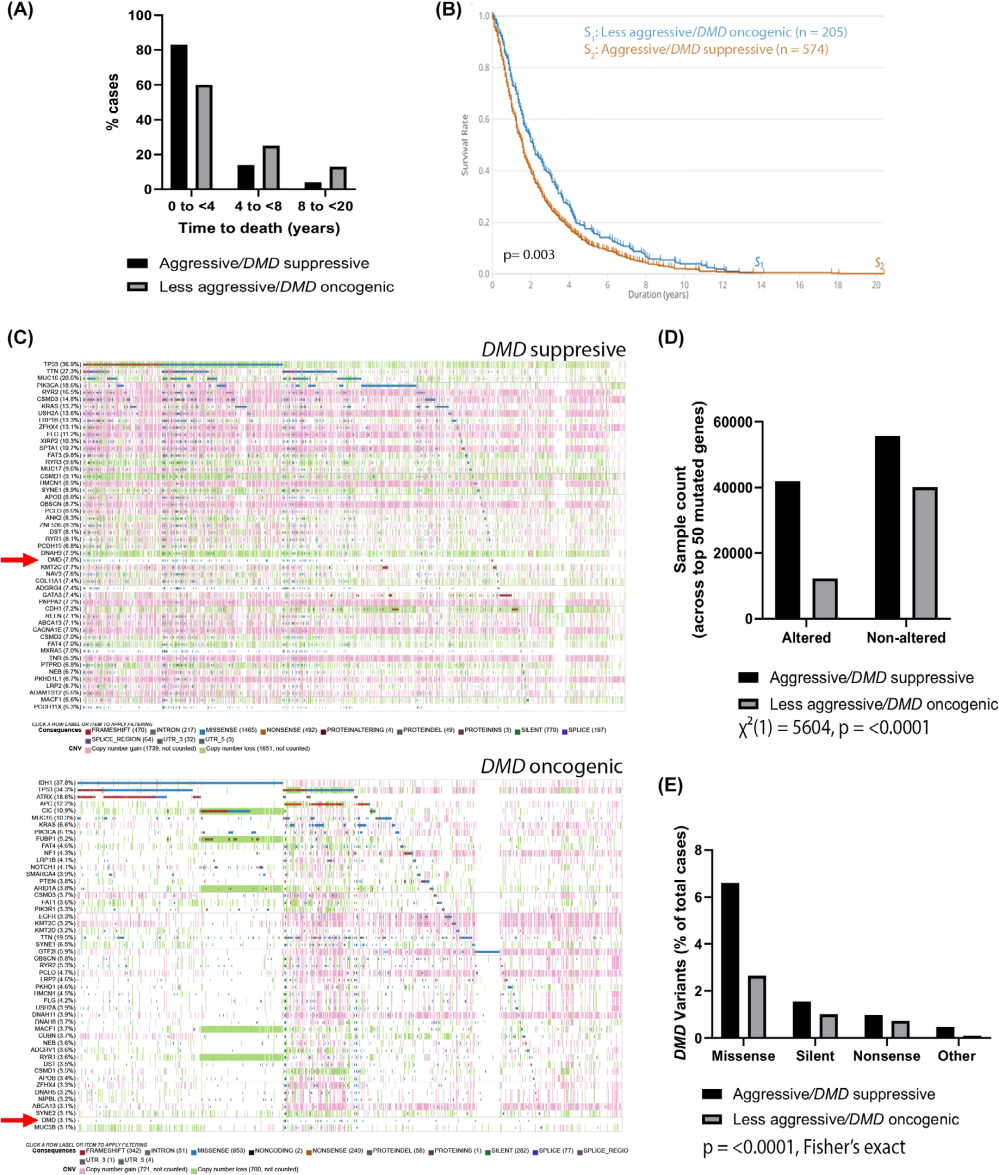
*DMD* acts as a tumour suppressor in more aggressive cancers and as an oncogene in less aggressive tumours. (A) Time to death distributions for the groups of tumours where high *DMD* is associated with better (*DMD* suppressive, *n* = 588) or worse (*DMD* oncogenic, *n* = 205) survival. A chi‐squared test (χ^2^(2) = 11.52, *P* = 0.0031) confirmed a significant difference between the two groups. (B) Overall survival for the proposed less aggressive/*DMD* oncogenic (*n* = 205) versus aggressive/*DMD* suppressive (*n* = 574) group, log rank test *P* value = 0.003. (C) Oncoplots of the *DMD* suppressive (top) and *DMD* oncogenic (bottom) tumour groups showing the top 50 mutated genes in each cohort. The position of *DMD* within each group is indicated by a red arrow. (D) Aggregated altered/unaltered sample counts across the top 50 genes for each group, χ^2^(1) = 5604, *P* < 0.0001. (E) The percentage of *DMD* gene alterations per group are shown, the distribution of alterations differs between the *DMD*‐suppressive and *DMD*‐oncogenic groups (*P* < 0.0001, Fisher's exact test on raw sample counts with/without alterations).

We first compared time to death and overall survival statistics between the aggressive versus less aggressive groups using the GDC Data Portal Cohort Builder and Analysis Center tools. A large majority of cases in the proposed aggressive group (83%) survived less than 4 years, compared to only 60% in the proposed less aggressive group (Fig. 6A). In the 4 to < 8‐year category, 14% of the aggressive group survived, compared to 25% in the less aggressive group. In the 8 to < 20‐year category, survival rates were 4% and 13%, respectively. A chi‐squared test (χ^2^(2) = 11.52, *P* = 0.0031) confirmed a statistically significant difference in time to death distributions between the two groups. This supports the classification of the *DMD* suppressor group as more aggressive/invasive, with shorter survival times compared to the *DMD* oncogenic group. Similarly, we compared overall survival across the two cohorts of tumours, which also showed a significant difference (*P* = 0.003, Fig. 6B). Since aggressive tumours often have a higher rate of genetic mutation [24], we used the GDC Analysis Center tools to also explore the alteration frequencies of the top 50 mutated genes in each cohort of tumours (Fig. 6C). There are more gene alterations (including copy number variations) in the *DMD* suppressive group than in the *DMD* oncogenic group (Fig. 6D; χ^2^(1) = 5604, *P* < 0.0001). Interestingly, *DMD* appears among the top 50 mutated genes in both groups at position 29 (altered in 7.8% of cases) in the *DMD* suppressive group and at position 49 (altered in 3.1% of cases) in the *DMD* oncogenic group (Fig. 6D [red arrows], 6E and Fig. S4). The distribution of *DMD* alterations significantly differs between the *DMD*‐suppressive and *DMD*‐oncogenic groups (Fig. 6D, *P* < 0.0001, Fisher's exact test on raw sample counts with/without alterations). A total of 206 *DMD* variants were identified from 153 samples (out of 2148 total cases) in the aggressive/*DMD*‐suppressive group (predominantly missense mutations) compared to only 49 *DMD* variants from 33 samples (out of 1093 total cases) in the less aggressive/*DMD*‐oncogenic group (Fig. 6E). Thus, these data support our hypothesis and the classification of the *DMD* suppressive tumour group as more aggressive than the *DMD* oncogenic tumour group. This highlights a complex relationship between *DMD* expression and tumour aggressiveness.

### Proposed model and evaluation

**Fig. 7:**
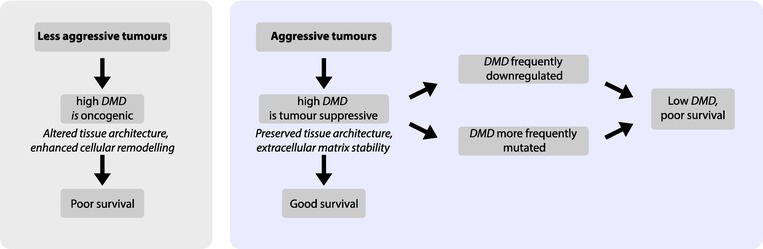
A unified model for *DMD* gene involvement in cancer. In less aggressive tumours high *DMD* expression leads to poor survival, whereas in aggressive tumours, high *DMD* expression is associated with better survival outcomes. However, in aggressive tumours, *DMD* is more frequently downregulated and/or mutated leading to low *DMD* expression and poor survival. We therefore propose that *DMD* can act as a tumour suppressor or an oncogene in a context‐dependent manner during tumorigenesis.

The distinct pathway enrichment profiles and our survival and alteration frequency analyses presented above support a context‐dependent role for *DMD* (and most notably, the Dp71ab gene product) in cancer, which reconciles conflicting reports of its oncogenic versus tumour‐suppressive functions. Based on these findings, we propose a unified model in which *DMD* acts as a tumour suppressor in aggressive cancers but exhibits oncogenic properties in less aggressive cancers (Fig. 7). For this model, we define aggressive cancers as those typically characterised by a combination of high metastatic potential, poor overall survival, frequent recurrence, resistance to therapy and a high burden of genetic alterations, with multiple factors typically present. Based on our findings here and the literature [2], we additionally theorise that *DMD* is more frequently mutated and/or downregulated in aggressive tumours (Fig. 7). To validate the model, we examined additional cancers known from the literature to also be significantly associated with *DMD* expression but that were not identified as significant in our bioinformatic analysis when stringently adjusting for multiple comparisons. These were sarcoma, head and neck squamous cell carcinoma (HNSCC) and mesothelioma. Based on previously published known survival associations, sarcoma and HNSCC belong to the *DMD*‐suppressor group, since high *DMD* expression is associated with improved survival [9, 12, 26], while mesothelioma aligns with the *DMD*‐oncogenic group, since high *DMD* expression correlates with poorer survival [7]. For our model to stand, sarcoma and HNSCC should be considered as aggressive, whilst our model posits that mesothelioma be less aggressive. Sarcoma and HNSCC are indeed highly aggressive and metastatic cancers with early and frequent distant spread [27, 28] supporting their categorisation into our more aggressive/*DMD* suppressive group (Table 1). Additionally, sarcoma (which has high genomic instability [29]) and head and neck cancer are also amongst the tumours with the highest number of *DMD* alterations [2] and HNSCC appears amongst the lists of tumours where *DMD* expression is significantly downregulated compared to control tissue [8]. HNSCC is recognised for therapy resistance and high recurrence rates, further supporting our model classification [30]. Moreover, in a recent focused study of *DMD* in HNSCC, we also revealed an upregulation of ECM structure and ECM organisation processes in *DMD* high versus low tumours, in alignment with the *DMD* suppressive group presented here [12]. Whilst mesothelioma has a poor prognosis, it remains largely confined to its site of origin, primarily spreading via local invasion rather than distant dissemination [31]. *DMD* alterations are also less frequent in mesothelioma, and it has a low tumour mutation burden [2, 32]. Given our model's definition of aggressive, mesothelioma can fit as a less aggressive tumour despite some inconsistencies (Table 1). Thus, our model for the first time successfully integrates conflicting findings on *DMD*'s oncogenic versus tumour‐suppressive behaviours, demonstrating that *DMD* behaves as a tumour suppressor in more characteristically metastatic (i.e. aggressive) cancers but exhibits oncogenic properties in less aggressive, locally invasive tumours (Fig. 7 and Table 1). These findings provide a unifying framework for interpreting the role of *DMD* and its gene products in cancer progression and lay the groundwork for further functional validation.

## Discussion

We comprehensively explored the prognostic significance of *DMD* and DAPC gene expression across TCGA cancer types. We identified nine cancers where high versus low *DMD* expression was significantly associated with overall survival and present a model for a context‐dependent dual role of *DMD* in cancer that reconciles previous conflicting reports. In aggressive tumours with high metastatic potential, low *DMD* expression correlates with poor survival, supporting a tumour suppressor function. Additionally, the higher frequency of *DMD* mutations in this group suggests a selective pressure for *DMD* loss in aggressive cancers, reinforcing its tumour‐suppressive role. Conversely, in less aggressive tumours, high *DMD* expression correlates with poor survival, suggesting an oncogenic role in these settings. Our study highlights Dp71ab as an important dystrophin variant influencing tumorigenesis and survival in alignment with total *DMD* survival trends. We also found that the relatively poorly characterised dystrophin isoform, Dp40, was expressed in all nine cancers. We present evidence that the role of *DMD* and dystrophin variants in cancer may be linked to a subset of signalling and adhesion‐related DAPC components, which cluster separately from the sarcoglycans, suggesting that *DMD* may influence tumour progression via signalling pathways rather than mechanical stability alone. Investigating the mechanistic roles of *DMD* gene product expression in driving tumour aggression and/or modulating oncogenic transcriptional programs was beyond the scope of this in silico study; this remains a critical knowledge gap that warrants a focused in‐depth investigation.

We tested our model, confirming its consistency with established findings in the literature. Our own prior work in LGG and HNSCC [10, 12] provides immunohistochemical validation, confirming dystrophin protein expression in both the nucleus and cytoplasm of tumour cells. Notably, a significant correlation between dystrophin protein expression and patient survival is observed, reinforcing survival trends seen at the RNA level and demonstrating the clinical relevance of dystrophin protein localisation in tumour biology. Our model is further supported by the observation that *DMD* survival trends differ between less and more aggressive cancers of the same tissue type. For instance, *DMD* expression is prognostic in low‐grade glioma (LGG) but not in the more aggressive glioblastoma [10].

Using a mathematical approach, Padder et al. [33] also recently developed a model that simulates interactions between dystrophin and tumour microenvironment components to predict how changes in dystrophin levels affect tumour growth and progression. Their model only focuses on tumours where downregulated dystrophin is correlated with reduced survival (i.e., the aggressive tumour group in our model). Their bifurcation analyses suggest that, in these tumours, the strength of feedback between dystrophin expression and tumour growth is a critical factor influencing stability. Their simulations, in line with our model, suggest that when dystrophin is lost, the tumour destabilises faster, pushing it towards uncontrolled growth, suggesting that dystrophin helps regulate tumour dynamics. Our model supports and extends this to show that *DMD* has both tumour‐suppressive and oncogenic roles, depending on the context. It unifies existing conflicting knowledge into a single model, addressing cancers where low *DMD* is harmful as well as those where low *DMD* is beneficial.

Whilst other *DMD* transcripts and dystrophin proteins may be relevant to tumourigenesis in some specific contexts [34], our work supports Dp71ab as a ubiquitously expressed key player in *DMD*‐associated cancers. Functionally, previous studies in sarcoma and HNSCC cell models have shown that Dp71ab overexpression leads to reduced proliferation [12, 26], aligning with our proposed tumour‐suppressive role for *DMD* in aggressive sarcomas and HNSCC. The ablation of Dp71 isoforms lacking exon 78 (collectively known as Dp71f) in sarcoma cell lines has also been shown to increase proliferation, enhance colony formation and disrupt ECM‐receptor interaction pathways [9], providing further support for our model. Though no significant changes in migration or invasion assays in this study were observed, there was an increase in the levels of matrix metalloproteases (MMP) 9 and 2 released into the media in Dp71f ablated cells compared to control. MMPs are recognised to facilitate tumour cell invasion and metastasis by degrading the ECM [35, 36].

While our findings provide strong support for a dual role of *DMD* in cancer, we acknowledge limitations. We have defined aggressiveness based on several factors including metastatic potential instead of just survival rates, but we recognise that labelling tumours simply as aggressive/less aggressive does not fully capture the complexity of tumour behaviour, which is influenced by molecular, genetic and environmental factors. Survival trends (and *DMD* transcript expression) may also not be uniform across all tumour subtypes and may be confounded by clinicopathological variables. Indeed, we have previously shown differences across IDH wild‐type and mutant LGG subtypes and HPV positive and negative HNSCC subtypes [10, 12] highlighting heterogeneity across tumour subtypes that our model does not necessarily capture. While functional validation now exists for several cancers [9, 10, 12, 26], more work is required to establish clear mechanisms and validate *DMD*/Dp71ab as a therapeutic target in physiologically relevant model systems.

Currently, most studies on *DMD*‐associated cancers have ignored the complex pattern of *DMD* gene product expression and the multiple isoforms of Dp71; our model is also not yet fully tested to this level of complexity. A current limitation of the field is the lack of suitable antibody reagents that distinguish the different Dp protein gene products expressed in cancer tissues. Nonetheless, there is precedent for genes to act as either a tumour suppressor or oncogene, depending on context. For example, full‐length isoforms of the transcription factor p63 (Tap63 isoforms) are tumour suppressive in some contexts, but the N‐terminally truncated ΔNp63 isoforms are oncogenic in others [37, 38, 39]. Whilst we are not implying there is a correlation of *DMD* with p63 status, our work provides robust evidence for a similar context‐dependent role for *DMD*, where its high expression may support tumour‐promoting processes in some cancers while reinforcing tumour‐suppressive mechanisms in others. Previously, we suggested that the ratio of Dp427 to Dp71, rather than their absolute levels, may be a key factor in disease progression [2]. While this balance may still play a role, our updated model expands on this by demonstrating that total *DMD* expression, in a context‐dependent manner, is also a critical determinant of tumour behaviour. This broader perspective integrates both gene product levels and tumour‐specific factors to better explain the dual role of *DMD* in cancer progression. As the field begins to move towards validating *DMD* and/or its gene products as therapeutic targets in cancer, it is vital to consider context such as tumour type/subtype, stage, and microenvironment to avoid unintended consequences.

## Conclusions

Our findings reinforce a nuanced role for *DMD* in tumorigenesis. In aggressive cancers, high *DMD* expression appears to preserve DAPC‐mediated signalling and adhesion, countering metastatic potential, whereas in less aggressive tumours, elevated *DMD* may drive oncogenic pathways through enhanced cellular remodelling. This duality offers a novel perspective with significant implications for developing targeted therapeutic strategies that must consider the distinct tumour context.

## Conflict of interest

The authors declare no conflict of interest.

## Author contributions

LM and KA conceived the project; LM conducted most data analyses and prepared the first draft of the manuscript. KA helped to conceive and supervised the project, contributed data analysis, and critically edited the manuscript. LJ, MN, and SD contributed to data analysis and critically edited the manuscript. All authors have read and approved the final version of the manuscript.

## Supporting information


**Fig. S1.** *DMD* expression is significantly associated with survival in specific tumour types.
**Fig. S2.** Hazard ratios of TCGA tumours expressing specific *DMD* gene products.
**Fig. S3.** Association of DAPC gene expression with hazard ratios in selected TCGA tumours.
**Fig. S4.** *DMD* mutation frequencies across the aggressive/*DMD* suppressive and less aggressive/DMD oncogenic groups.

## Data Availability

The data that support the findings of this study are openly available in the TCGA portal (https://portal.gdc.cancer.gov/). No additional datasets were used or created for this study.
