# Supplementary material for: A unified model for Duchenne muscular dystrophy gene involvement in cancer: context‐dependent tumour suppression and oncogenicity
Source: FEBS Open Bio. 2025 Aug 20;16(1):222–36. doi: 10.1002/2211-5463.70109 (PMC12767769; doi:10.1002/2211-5463.70109)
Supplement: Supplementary file 1 — Fig. S1. DMD expression is significantly associated with survival in specific tumour types. Fig. S2. Hazard ratios of TCGA tumours expressing specific DMD gene products. Fig. S3. Association of DAPC gene expression with hazard ratios in selected TCGA tumours. Fig. S4. DMD mutation frequencies across the aggressive/DMD suppressive and less aggressive/DMD oncogenic groups. [file FEB4-16-222-s001.docx]

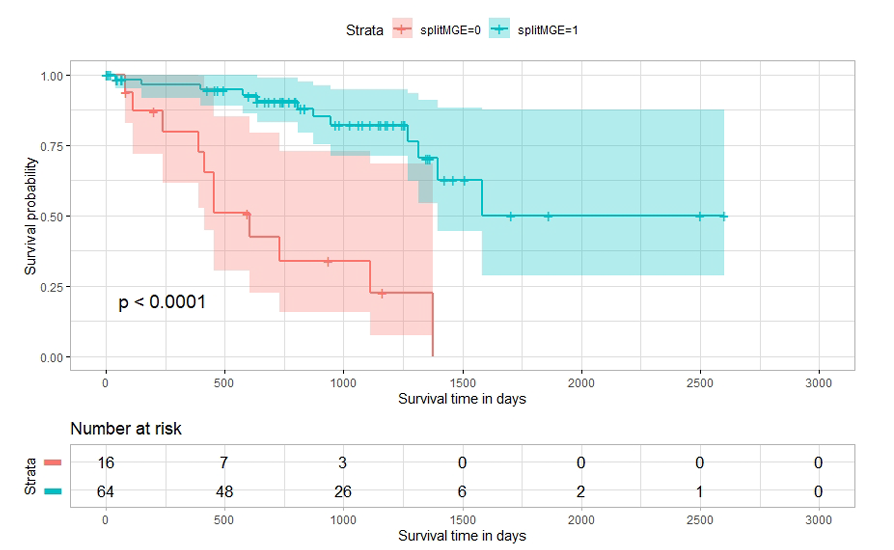

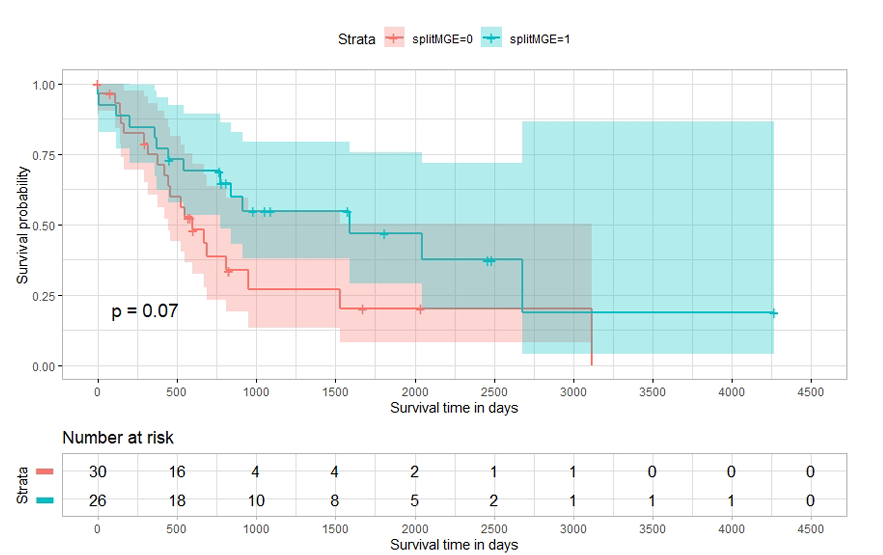

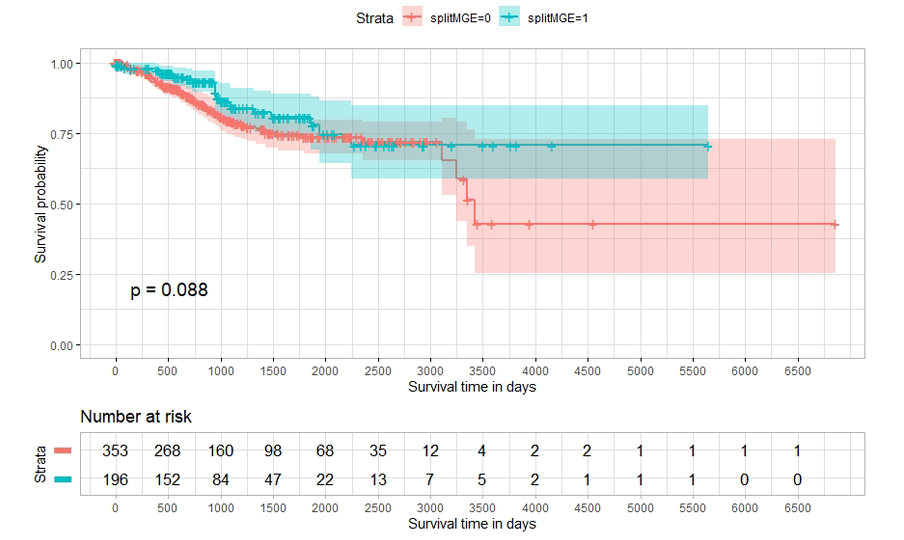

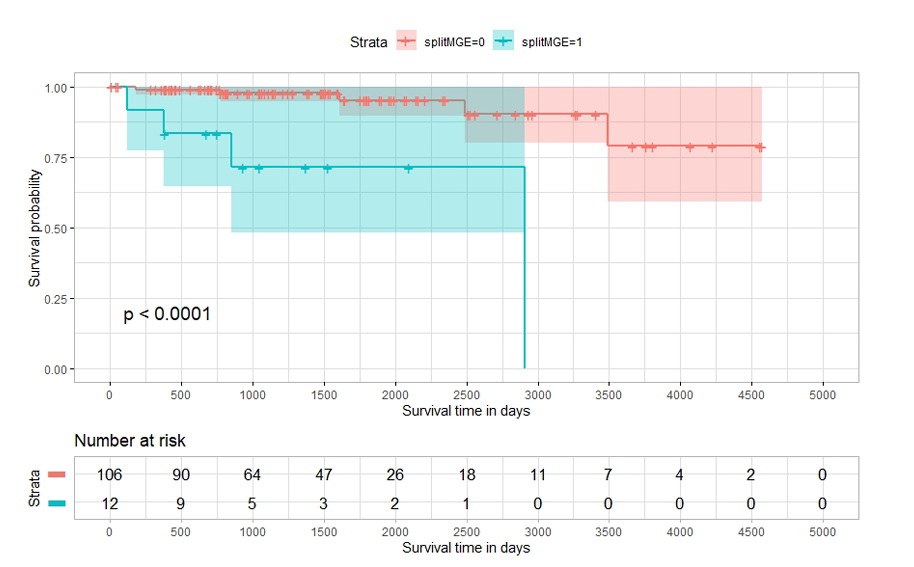

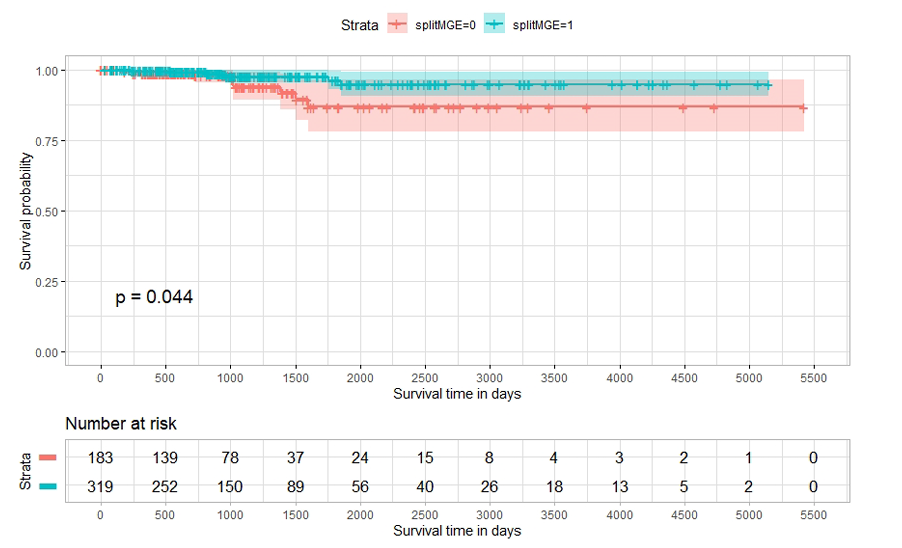

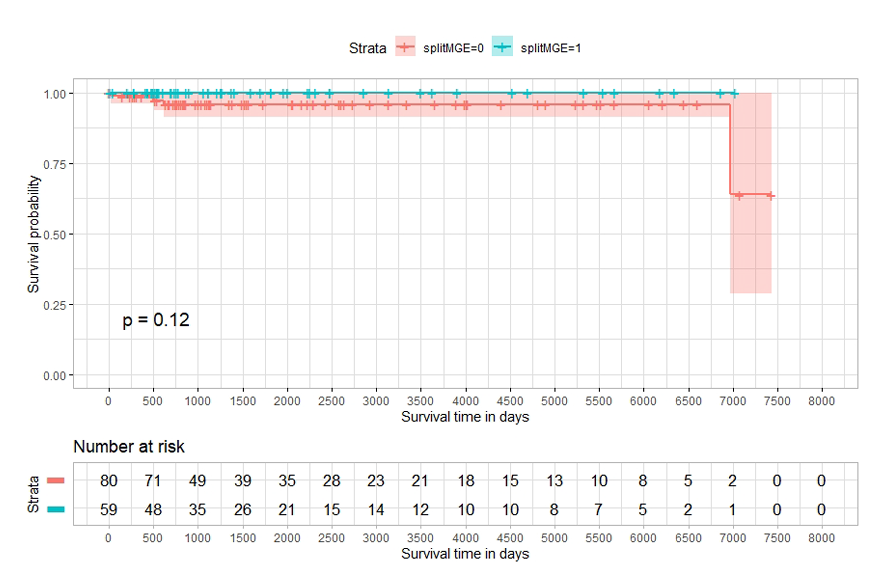

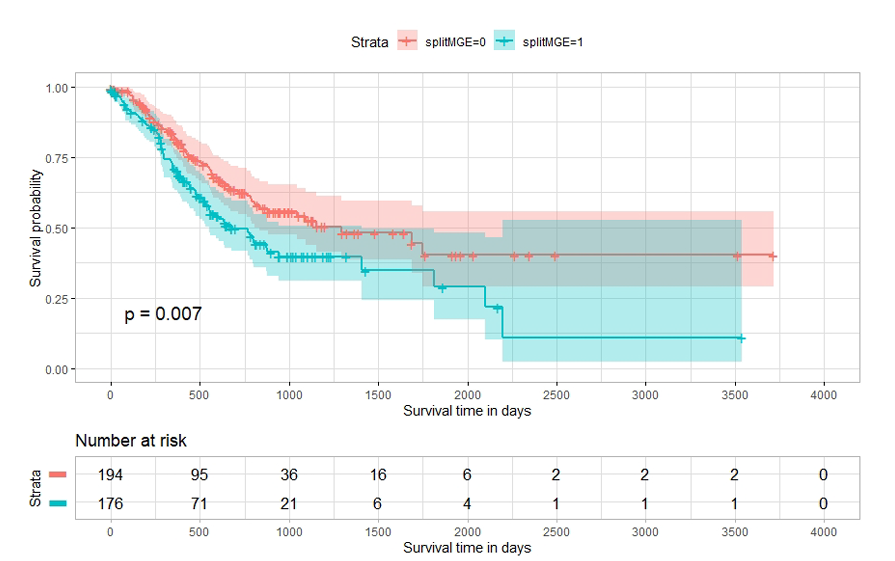

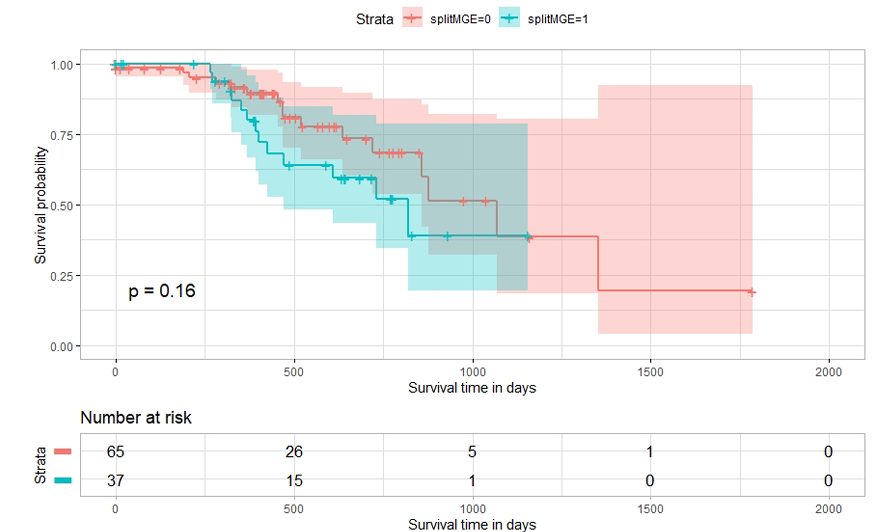

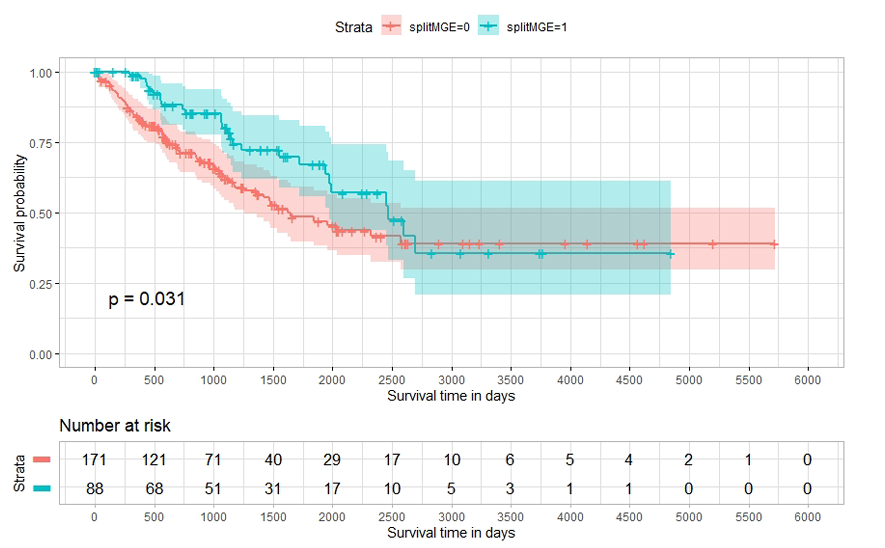

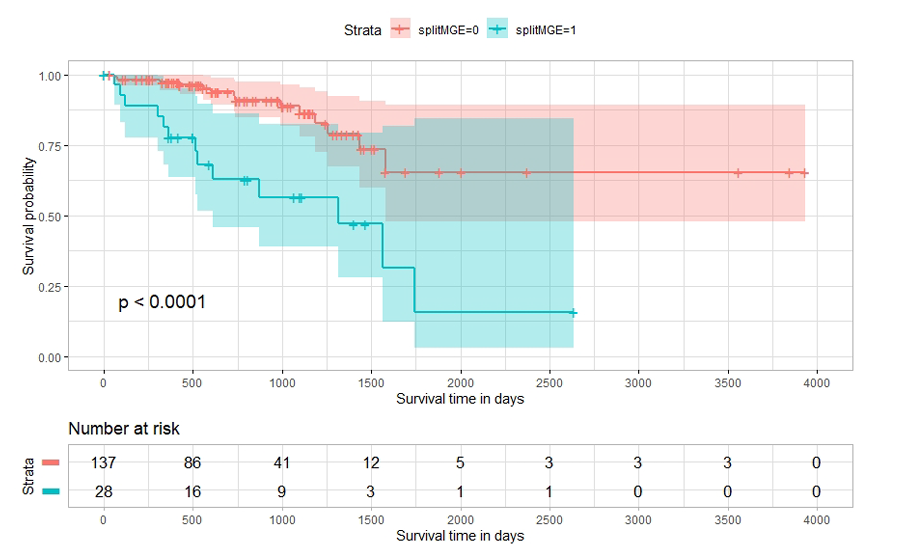

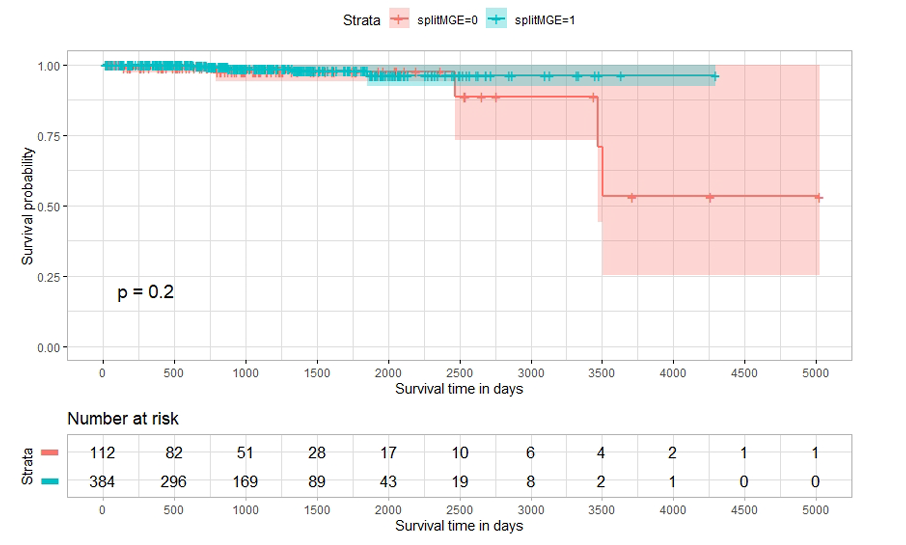

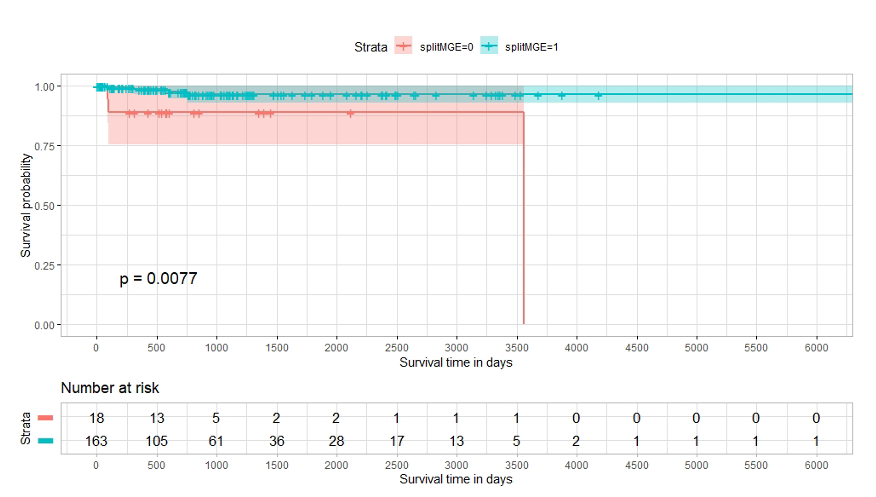

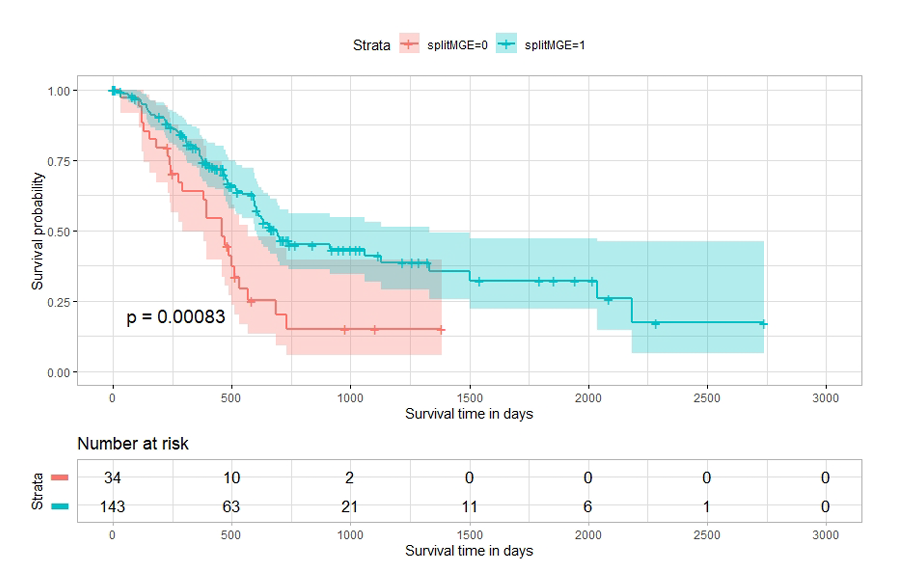

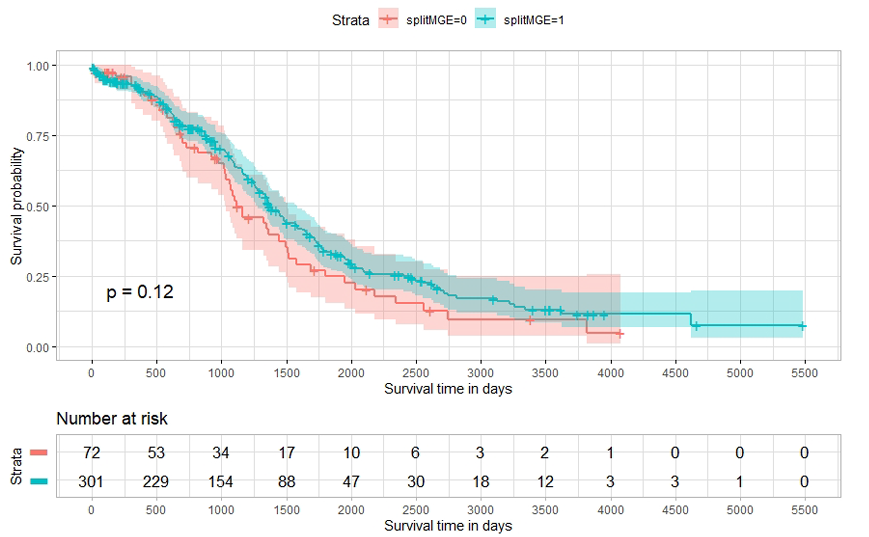

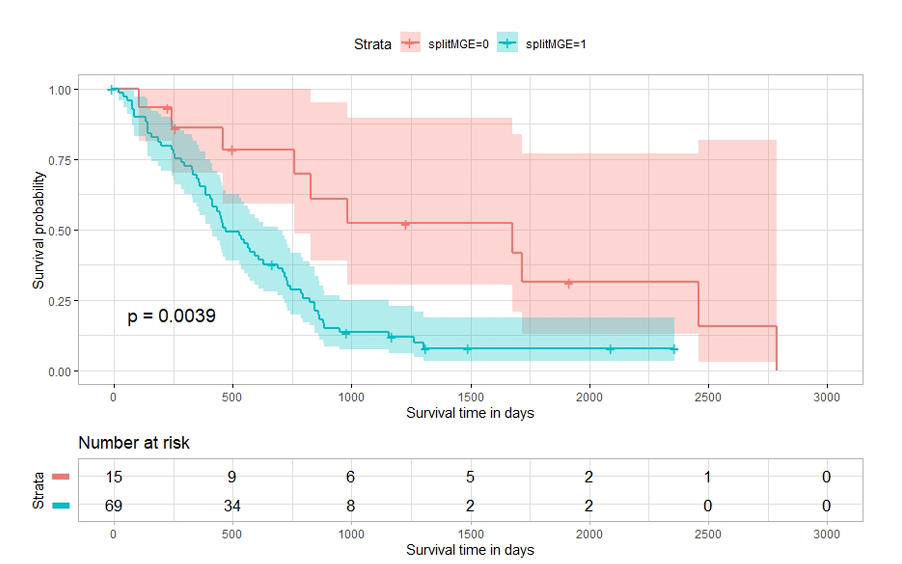

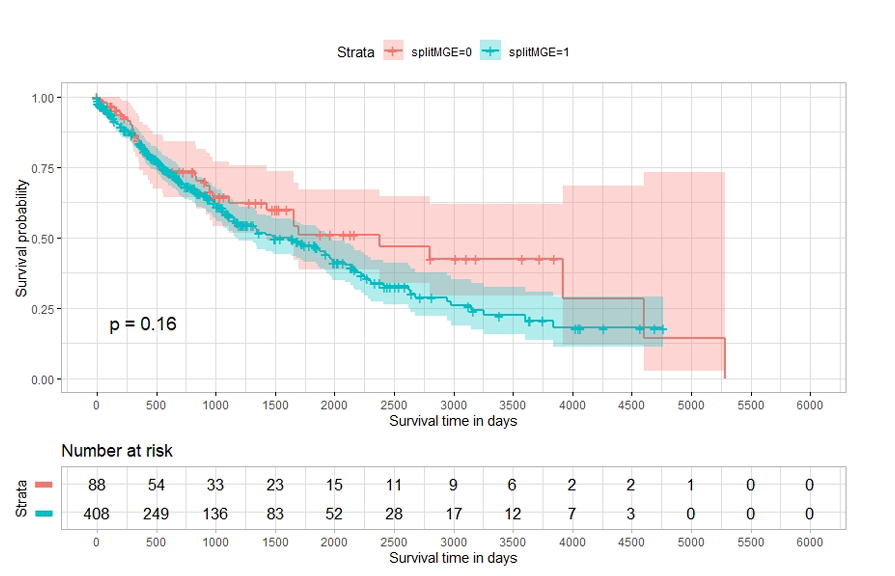

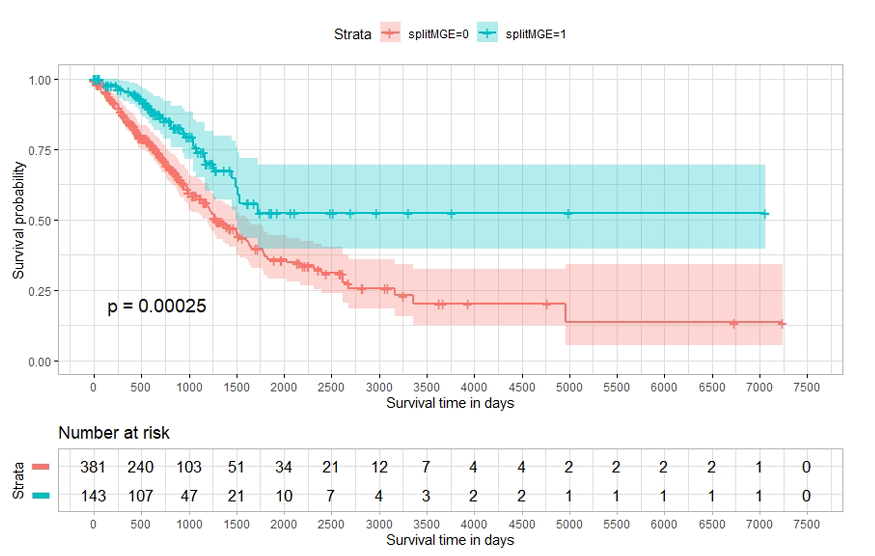

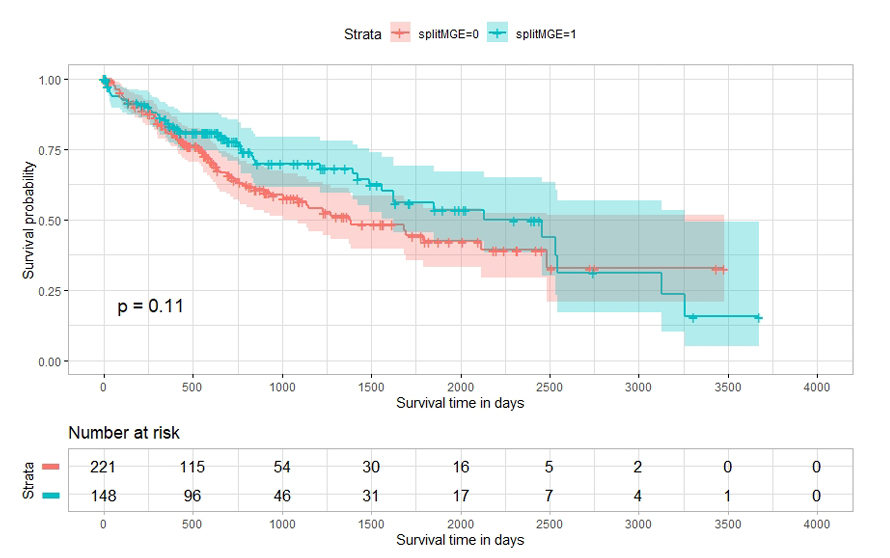

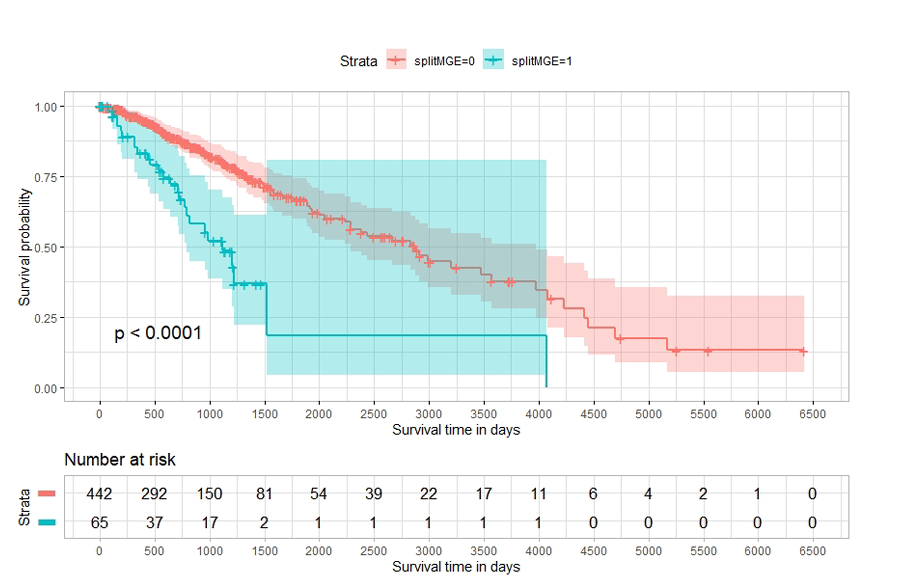

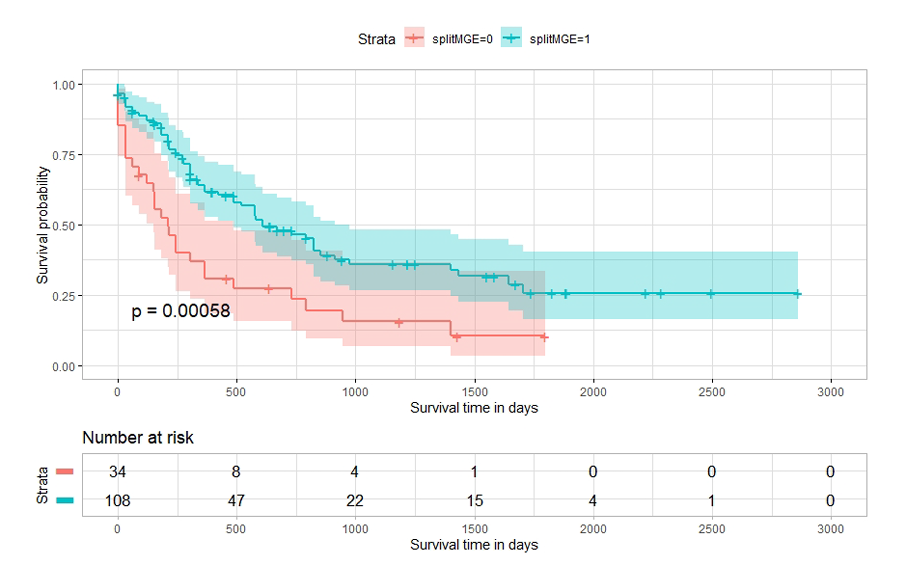

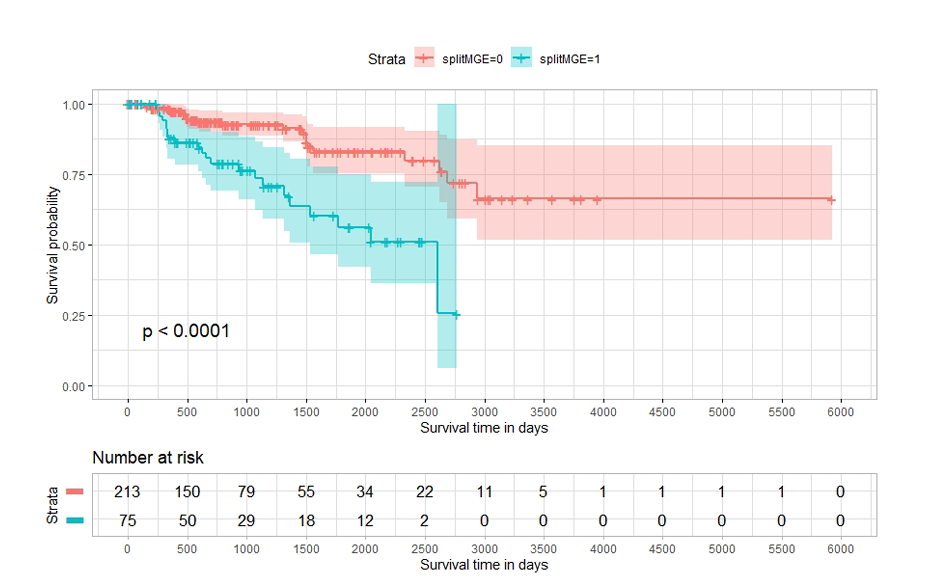

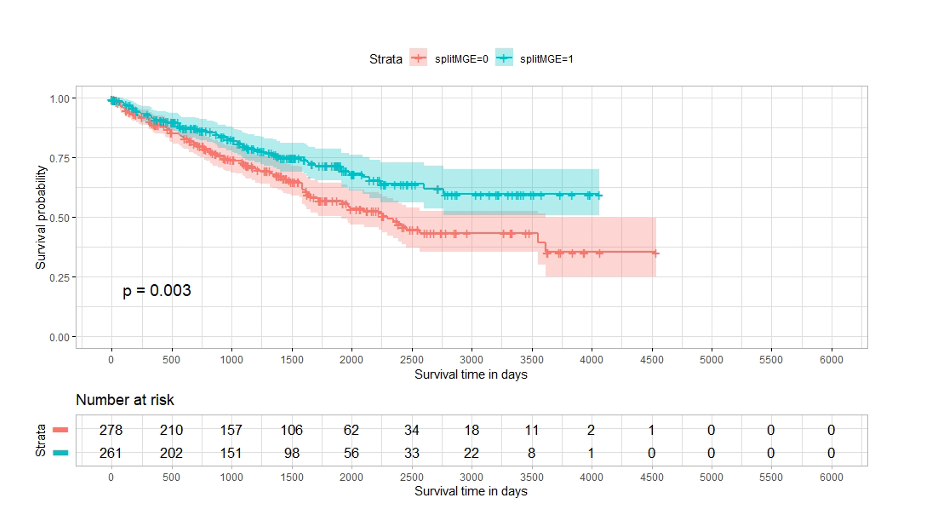

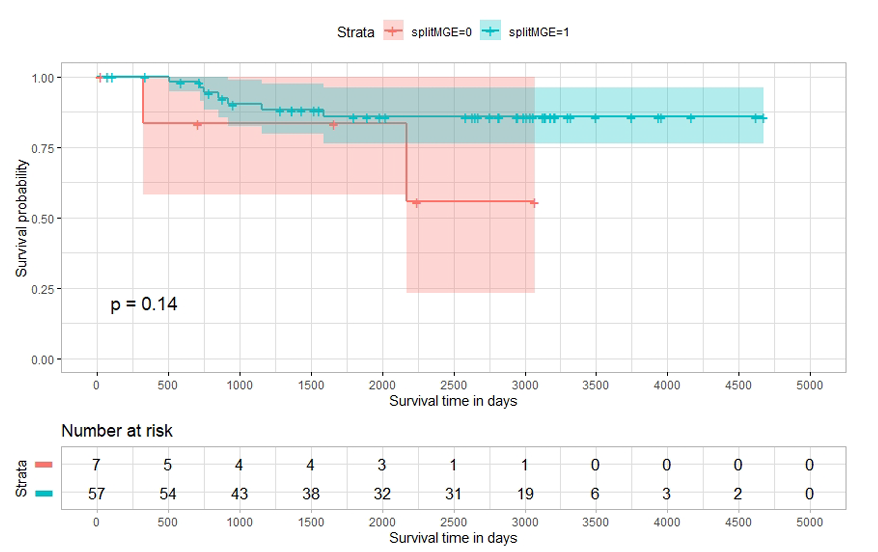

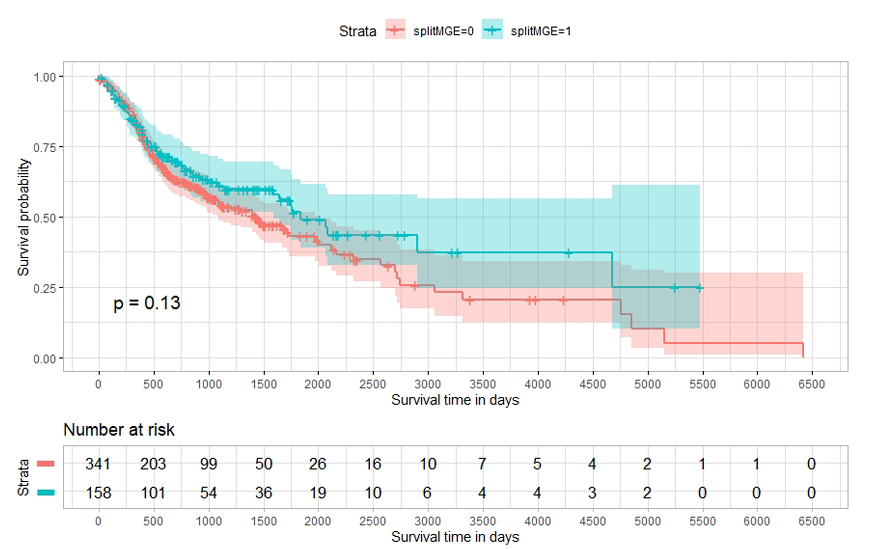

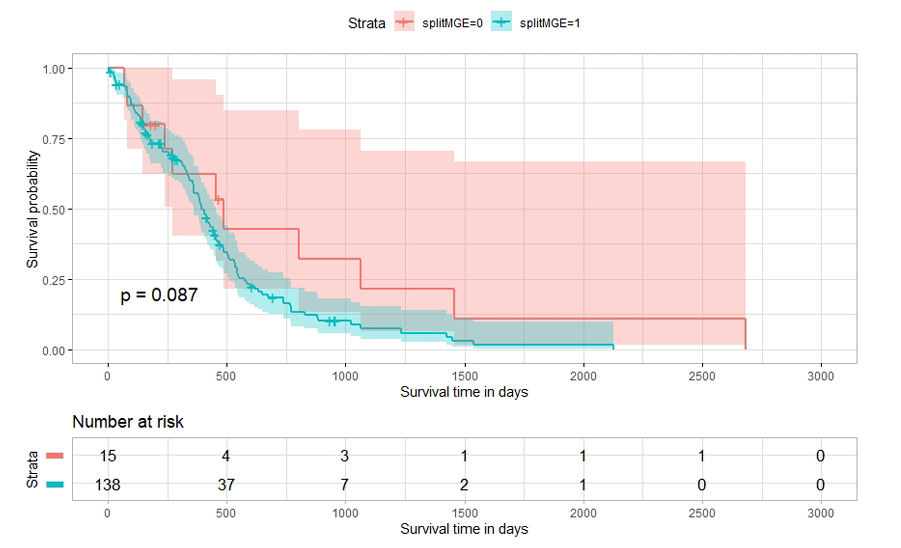

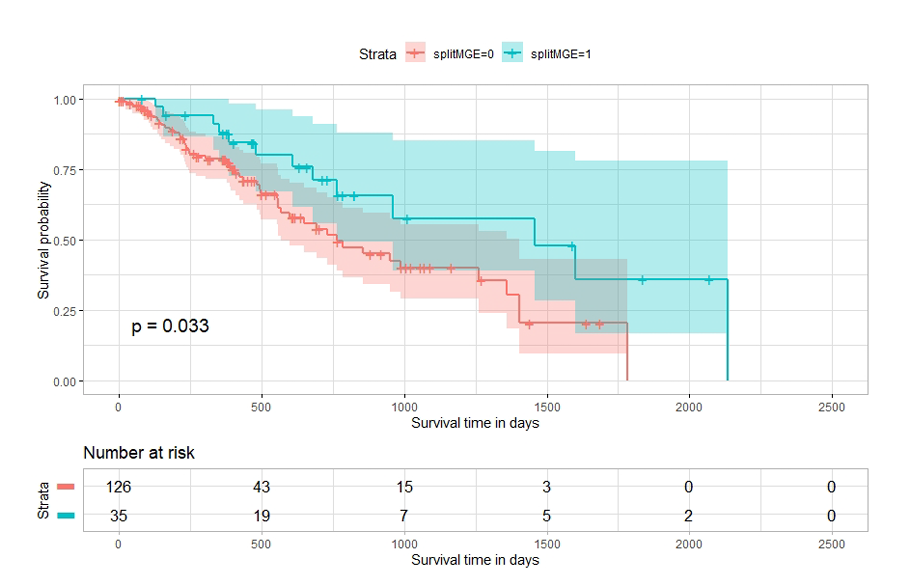

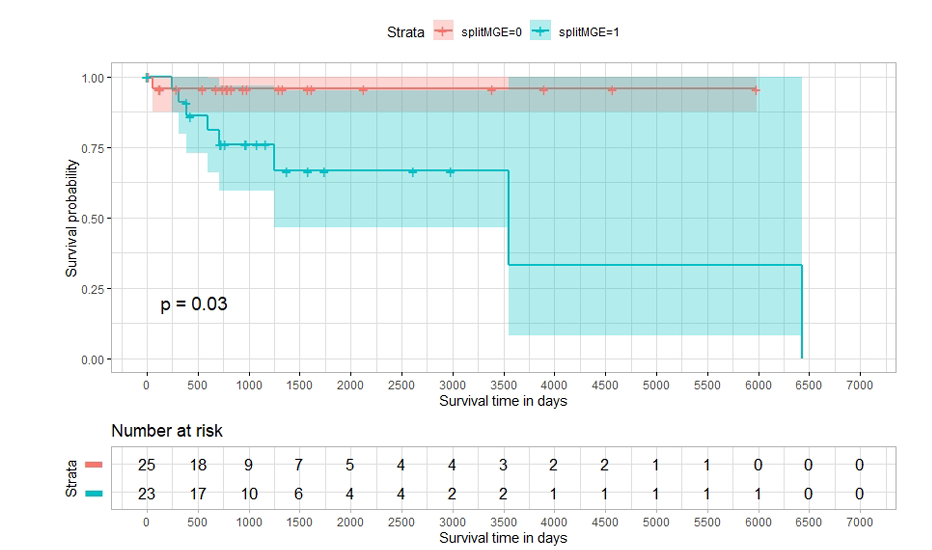

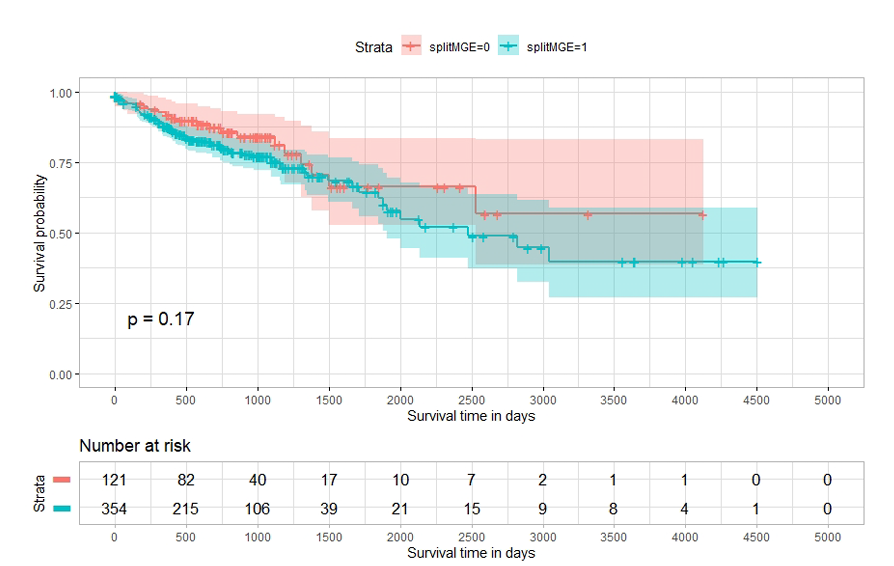

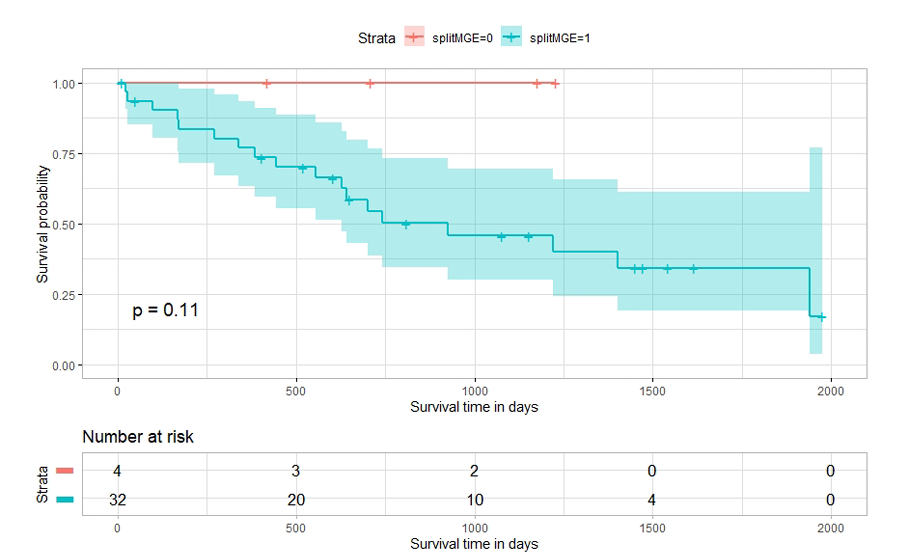

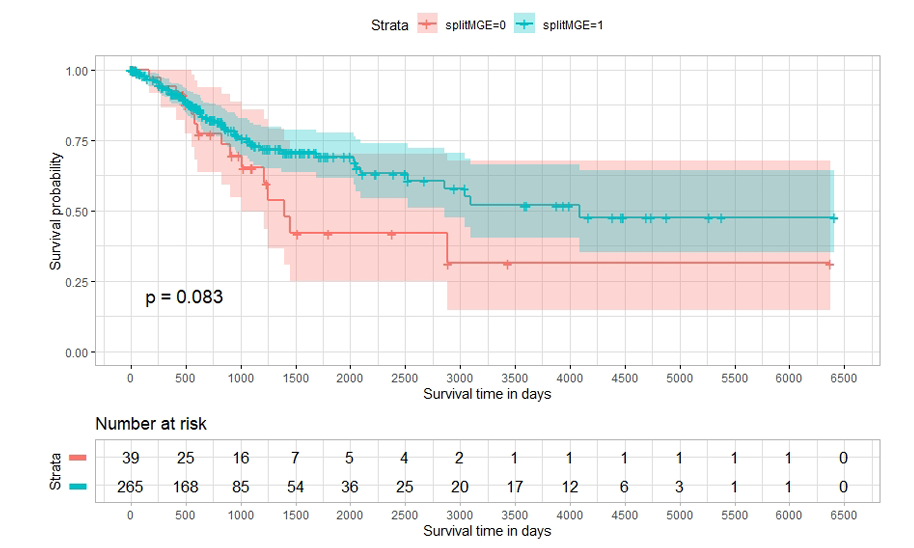

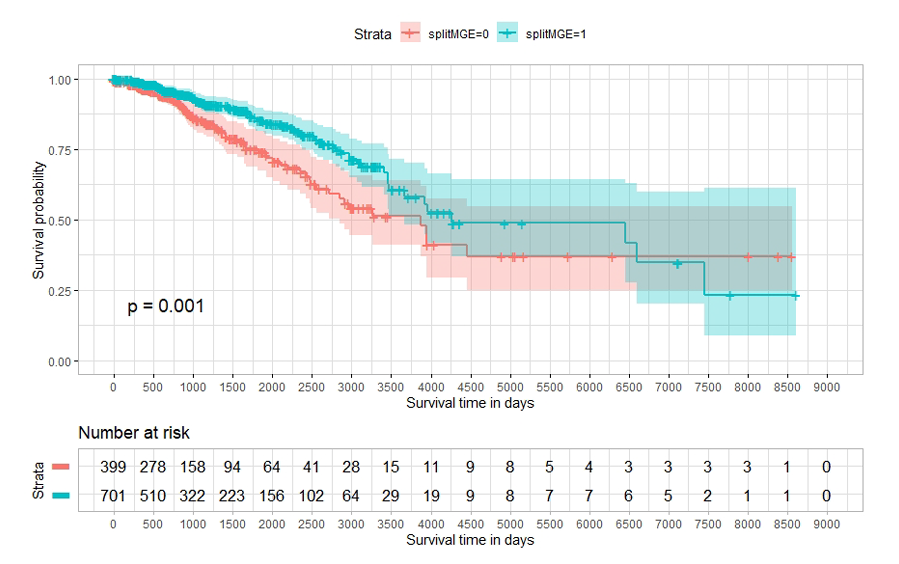

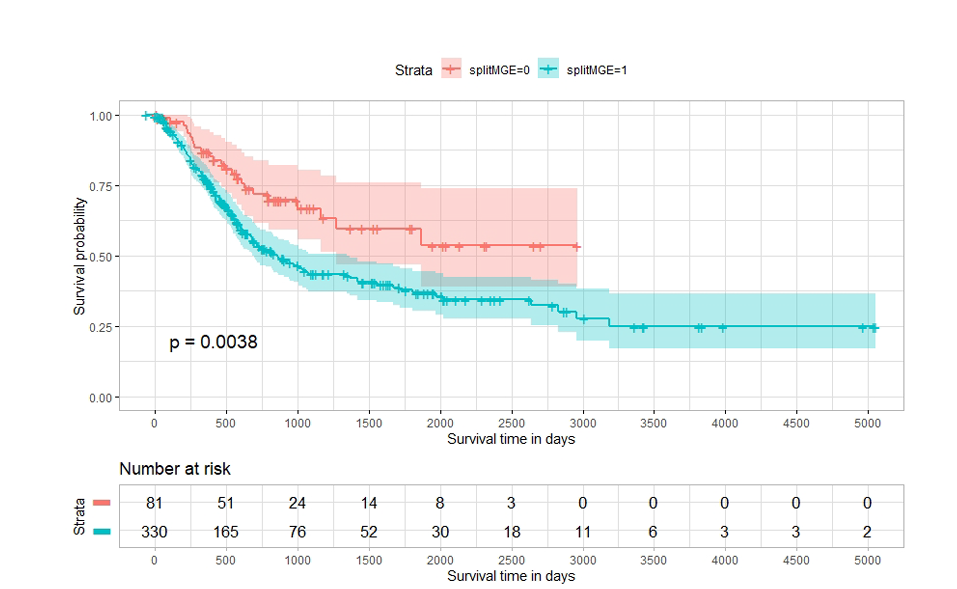

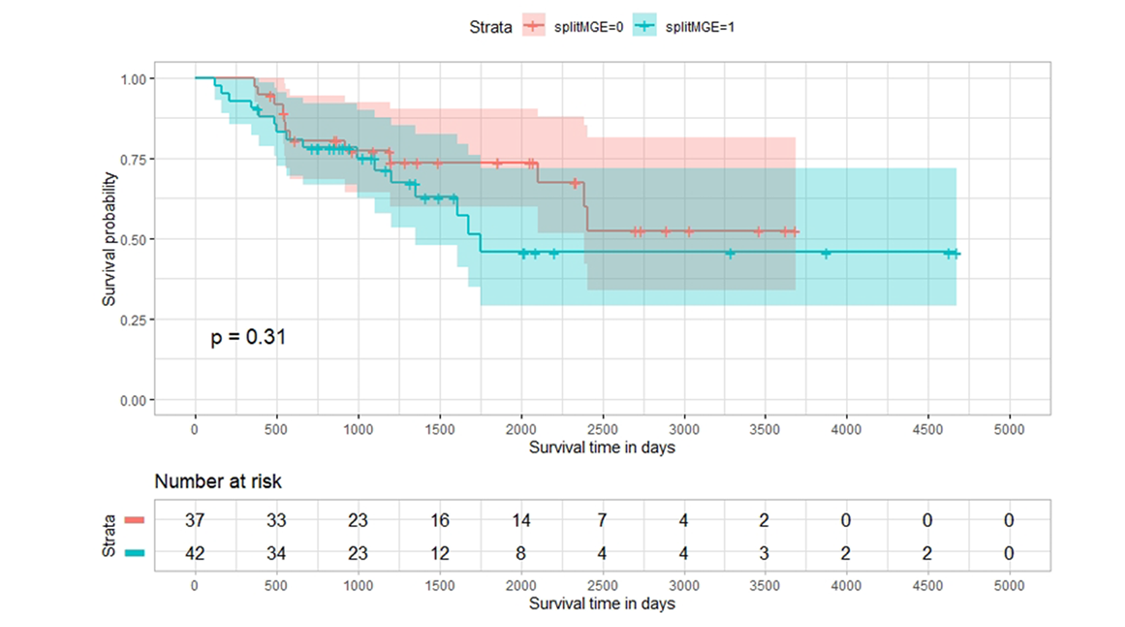


**UVM**

**UCS**

**UCEC**

**THYM**

**THCA**

**TGCT**

**STAD**

**SKCM**

**SARC**

**READ**

**PRAD**

**PCPG**

**PAAD**

**OV**

**MESO**

**LUSC**

**LUAD**

**LIHC**

**LGG**

**LAML**

**KIRP**

**KIRC**

**KICH**

**HNSCC**

**GBM**

**ESCA**

**DLBCL**

**COAD**

**CHOL**

**CESC**

**BRCA**

**BLCA**

**ACC**

Fig. S1. *DMD* expression is significantly associated with survival in specific tumour types TCGA RNAseq data from 33 TCGA cancer cases. Kaplan-Meier survival curves (overall survival) for high vs. low *DMD* expression. Red represents the low expression group and green represents the high expressing group. Uncorrected P values were calculated using the log-rank test. Survival analysis was performed in R. Risk tables indicate the numbers of patients in each group during observation


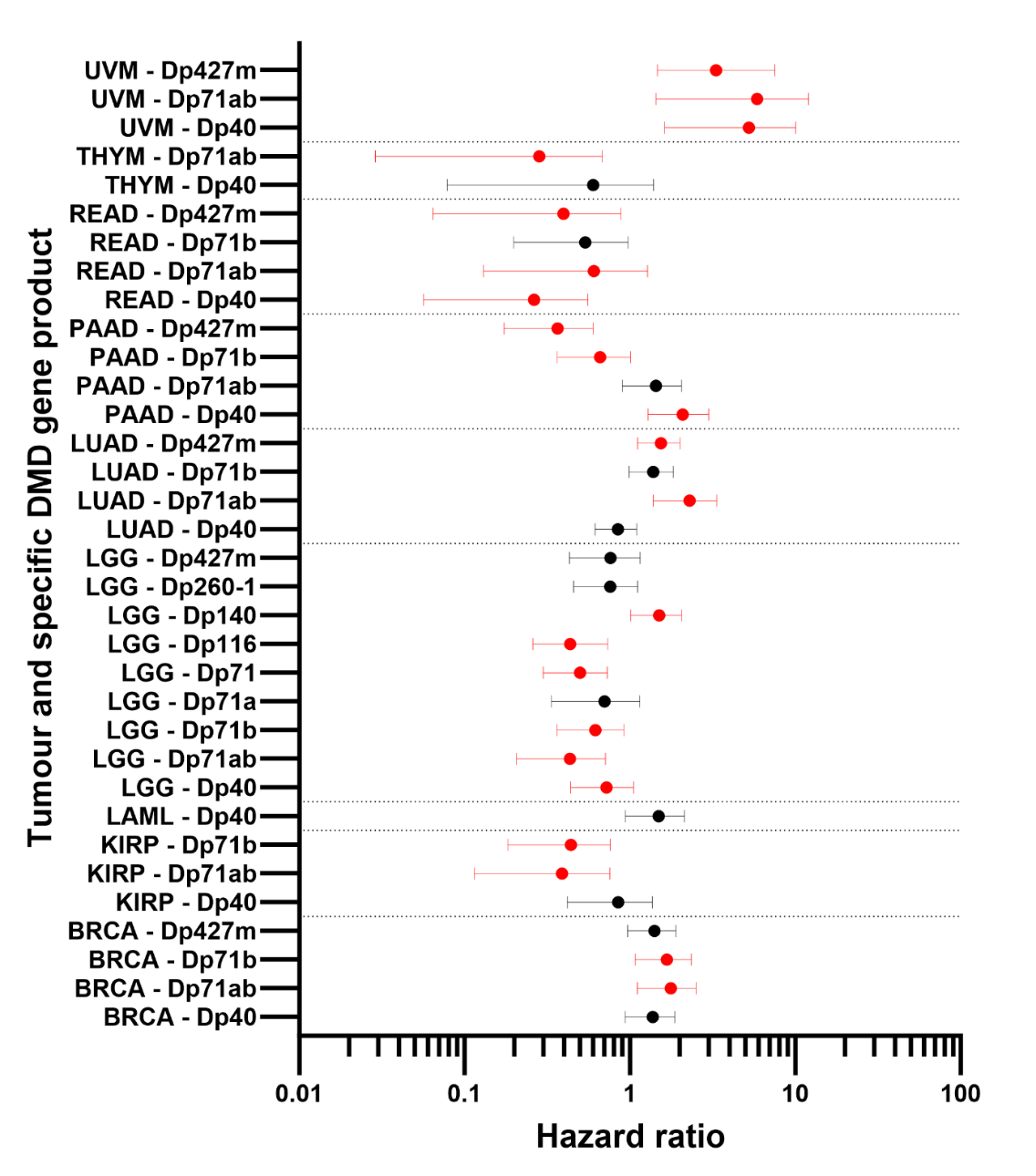


Fig. S2. Hazard ratios of TCGA tumours expressing specific *DMD* gene products. The *DMD* transcripts that are expressed in each tumour were analysed, the exact complement of transcripts differs according to tumour type. Forest plots revealing the log-rank hazard ratio with 95% confidence intervals. Red indicates significance (alpha < 0.05) UVM n=80, THYM n=121, READ n=177, PAAD n = 182, LUAD n=594, LGG n= 529, LAML n= 151, KIRP n= 321, BRCA n=1222


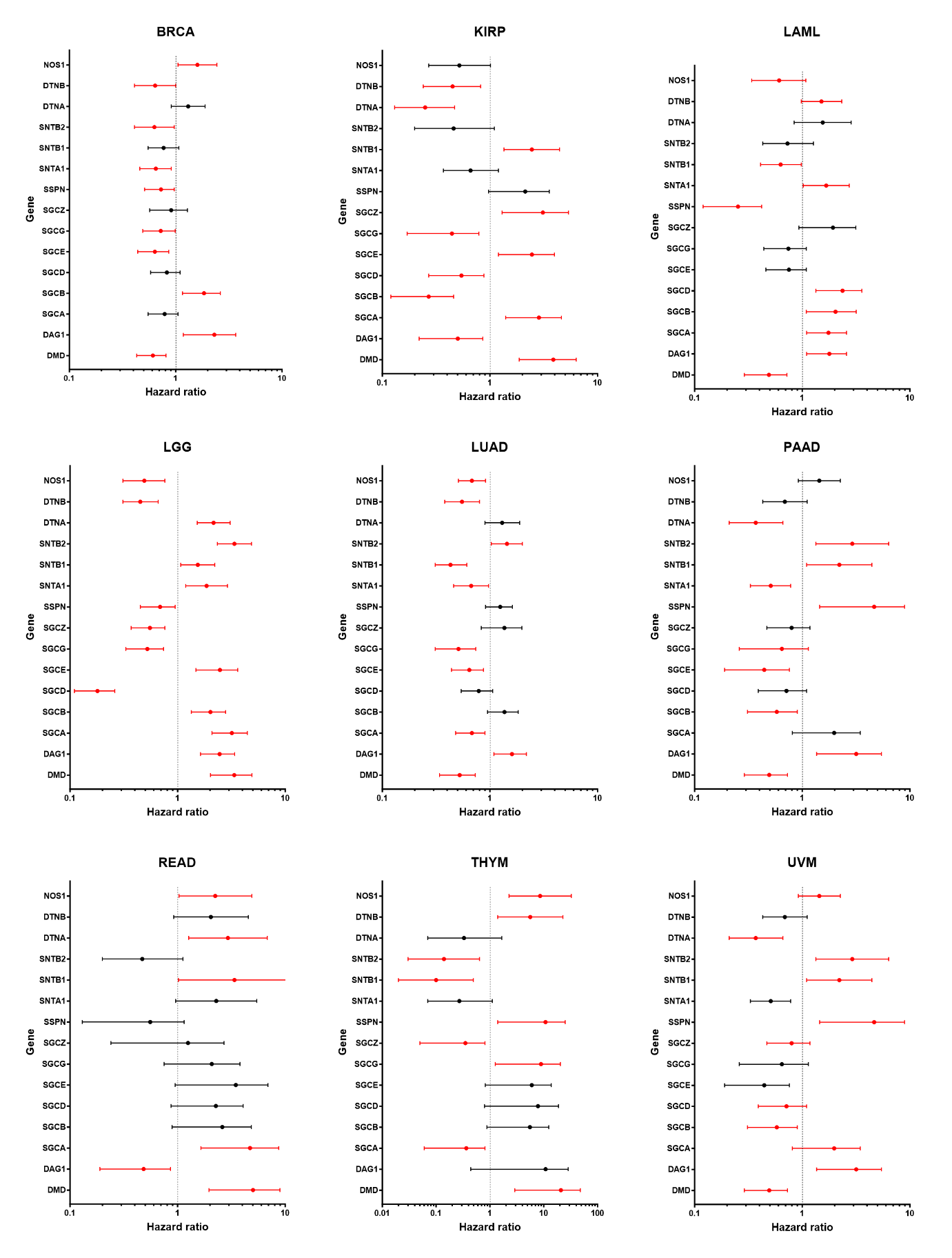
Fig. S3. Association of DAPC gene expression with hazard ratios in selected TCGA tumours. Forest plots revealing the log-rank hazard ratio with 95% confidence intervals. Red indicates significance (alpha 0.05). UVM n=80, THYM n=121, READ n=177, PAAD n = 182, LUAD n=594, LGG n= 529, LAML n= 151, KIRP n= 321, BRCA n=1222

**a Less aggressive/*DMD* oncogenic group**

**
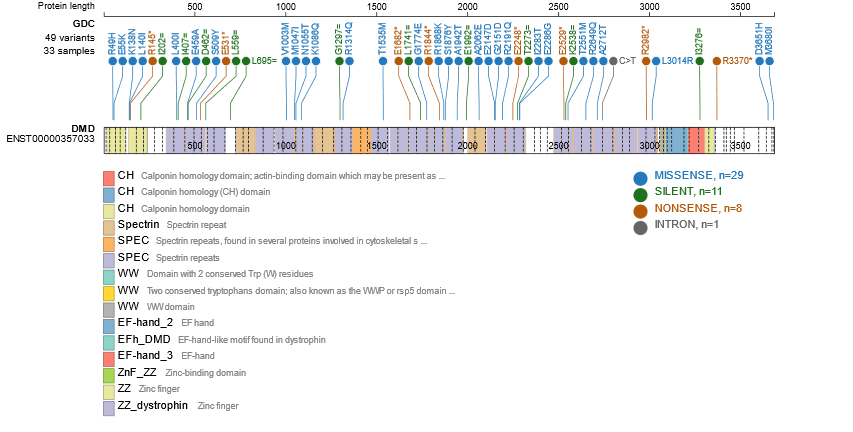
**

**b Aggressive/*DMD* suppressive group**


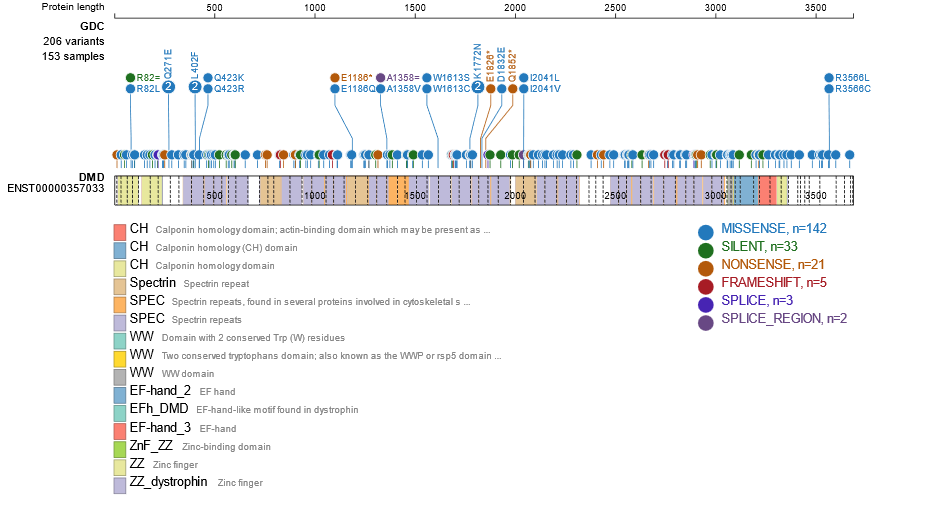


**Fig. S4.** ***DMD* mutation frequencies across the aggressive/*DMD* suppressive and less aggressive/*DMD* oncogenic groups.** (a) Mutations on the *DMD* gene within the less aggressive/*DMD* oncogenic group. (b) Mutations on the *DMD* gene within the aggressive/*DMD* suppressive group. The types of mutation and their positions within functional protein domains are illustrated.
